# Surface-Immobilized Photoinitiators for Light Induced Polymerization and Coupling Reactions

**DOI:** 10.3390/polym14030608

**Published:** 2022-02-04

**Authors:** Matthias Mueller, Christine Bandl, Wolfgang Kern

**Affiliations:** 1 Montanuniversitaet Leoben, Institute of Chemistry of Polymeric Materials, Otto-Glöckel-Straße 2, A-8700 Leoben, Austria; christine.bandl@unileoben.ac.at (C.B.); Wolfgang.Kern@unileoben.ac.at (W.K.); 2 Polymer Competence Center Leoben GmbH, Rosegger-Strasse 12, A-8700 Leoben, Austria

**Keywords:** immobilized photoinitiator, surface initiated photopolymerization, grafting from, grafting to, surface coupled initiator, photocoupling, coating, photoiniferter

## Abstract

Straightforward and versatile surface modification, functionalization and coating have become a significant topic in material sciences. While physical modification suffers from severe drawbacks, such as insufficient stability, chemical induced grafting processes efficiently modify organic and inorganic materials and surfaces due to covalent linkage. These processes include the “grafting from” method, where polymer chains are directly grown from the surface in terms of a surface-initiated polymerization and the “grafting to” method where a preformed (macro)-molecule is introduced to a preliminary treated surface via a coupling reaction. Both methods require an initiating species that is immobilized at the surface and can be triggered either by heat or light, whereas light induced processes have recently received increasing interest. Therefore, a major challenge is the ongoing search for suitable anchor moieties that provide covalent linkage to the surface and include initiators for surface-initiated polymerization and coupling reactions, respectively. This review containing 205 references provides an overview on photoinitiators which are covalently coupled to different surfaces, and are utilized for subsequent photopolymerizations and photocoupling reactions. An emphasis is placed on the coupling strategies for different surfaces, including oxides, metals, and cellulosic materials, with a focus on surface coupled free radical photoinitiators (type I and type II). Furthermore, the concept of surface initiation mediated by photoiniferters (PIMP) is reviewed. Regarding controlled radical polymerization from surfaces, a large section of the paper reviews surface-tethered co-initiators, ATRP initiators, and RAFT agents. In combination with photoinitiators or photoredox catalysts, these compounds are employed for surface initiated photopolymerizations. Moreover, examples for coupled photoacids and photoacid generators are presented. Another large section of the article reviews photocoupling and photoclick techniques. Here, the focus is set on light sensitive groups, such as organic azides, tetrazoles and diazirines, which have proven useful in biochemistry, composite technology and many other fields.

## 1. Introduction

Surface modification, functionalization and coating is of great interest both in academia as well as in the industrial sector. Among other aspects, the importance is related to the control of wettability and protein binding, to produce antifouling surface properties [1] or to achieve adhesive or anti-adhesive behavior of surfaces [2].

**Figure 1:**
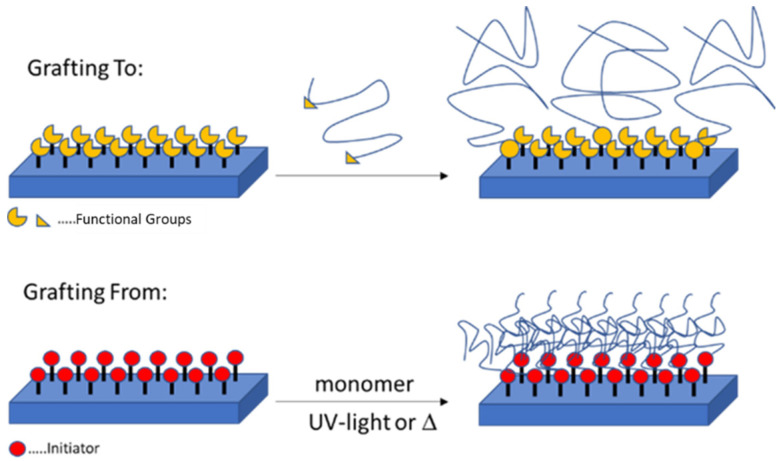
Schematic illustration of the “grafting from” and the “grafting to” approach (modified from ref. [8]). Blue lines indicate grafted polymer chains.

Generally, surface modification can be done by both, physical and chemical modification, whereas physical modification methods, such as spin casting or dip coating, have numerous disadvantages. These include, low thermal stability of the prepared films, their inability to withstand high shear forces and the fact that these layers can easily be removed by chemicals since they are only physically adsorbed to the substrate [1,3]. Chemical modification, on the other hand, is more desirable because it leads to more stable systems, which are covalently bound to the substrate surface and can be generated by two main grafting processes as shown in Figure 1. The “grafting to” approach is characterized by coupling a molecule (marked as blue line in Figure 1) with the desired properties onto the substrate surface. This can be achieved via the reaction between functional groups, one of which is tethered to the surface, or via photochemical reactions, such as addition or insertion reactions, employing a light sensitive species immobilized at the surface. Speaking of grafting polymers to substrates or rather of developing ultrathin polymer coatings or polymer brushes, this method suffers from severe limitations. Above all, steric hindrance between the macromolecular chains makes it difficult to covalently tether them next to each other in a densely-packed manner. Furthermore, with increasing molecular weight, the coupling-reaction between the functional (end)-group of the polymer and the complementary group on the substrate surface becomes less efficient. The “grafting from” method on the other hand, which can be considered as a bottom-up approach, directly initiates and therefore starts polymerization from the substrate surface. This is based on the light or heat triggered formation of a highly reactive species such as radicals derived from surface coupled initiators, which are able to start the polymerization mostly via addition reactions to multiple bonds. Compared to the “grafting to” mentioned approach, grafting from polymerizations leads to higher grafting densities and film thicknesses [1,4,5,6,7].

Over the last years, light induced processes have gained much interest because they exhibit important advantages over their thermal counterparts. These include fast reaction rates, low processing costs and easy exploitation of industrialization [1]. Further advantages are the spatial and temporal control of the triggered processes through the variation of radiation time, wavelength, intensity and beam focus [5]. Photopolymerization methods have found widespread application in industry, e.g., for the production of adhesives, coatings, inks and ultrafast drying varnishes [9]. Furthermore, photopolymerization-based 3D printing techniques, such as stereolithography, digital light processing or continuous liquid interface production, allow for the production of complex material systems with adjustable mechanical, optical and chemical properties. Recent progress and future trends in this field can be found in several references (e.g., [10,11]).

The large number of achievements in light mediated polymerization techniques also demonstrates their relevance for practical applications. For example, oxygen tolerance allows for a reduction of experimental effort since the process can be translated from inert-atmosphere to ambient conditions, thereby reducing cost and allowing for the implementation in high-throughput screenings [12]. Density functional theory (DFT) is a powerful tool to unveil detailed mechanisms, as it has been used exemplarily for the decarboxylation mechanism of a thioxanthone chromophore upon light irradiation [13]. Nowadays, many photoinitiators are available, covering a broad absorption range, reaching from UV-C to the visible light area (many of them are listed in ref. [11], including their specific absorbance wavelength). Visible light absorbing initiators have gained much interest recently, especially for biomedical applications. In addition to a reduced risk of eye damage, visible light is less harmful to living cells and does not lead to generation of ozone [11]. Achievements have also been made in the field of photoredox catalysts, allowing for low catalysts loading (ppm range) during polymerization. However, many of them contain metals, thereby limiting the field of application due to potential metal contamination. This drawback was overcome by the invention of non-toxic organic photoredox catalysts, thus leading to metal-free photopolymerization techniques [14]. Advanced methods also include two-photon induced photopolymerization (TPIP), which requires specific photoinitiators capable of two-photon absorption (TPA). Here, photopolymerization only proceeds in the focus of a laser where the photon density is high enough to excite the initiator by TPA. TPIP is an advanced microfabrication method, carried out by using near infrared lasers, which is capable of producing 3-dimensional structures with spatial resolution down to 100 nm [15,16,17,18].

Surface initiated (photo)-polymerization (“grafting from”) as well as the “grafting to” counterpart are versatile techniques to easily modify surfaces of different materials. Polymer brushes, depending on their structure, thickness, density and architecture, provide a huge variety of surface functionalities such as wettability, stimulus responsive features, anti-fouling, protein binding, friction control, drug delivery, etc. [1,4,7,8,19,20,21,22] However, it is important to keep in mind that a surface coupled (photo)-initiating species is crucial for these chemical modification techniques. 

As a continuing study to the review of Sangermano (ref. [1]), this review aims to provide an overview on photoinitiators, which are covalently coupled to surfaces of different materials. In addition, some examples of immobilized (co)-initiating species, which trigger light mediated polymerizations in the presence of photoredox catalysts or photoinitiators are also considered. An emphasis will be placed on the introduction of the corresponding functional group to the surface. Details about well-known coupling strategies, such as silanization of (pretreated) oxidized surfaces and the formation of self-assembled-monolayers (SAM) from thiol compounds on gold surfaces, can be found in another comprehensive review (see ref. [23]). 

## 2. Coupled Type I and II Photoinitiators

**Figure 2:**
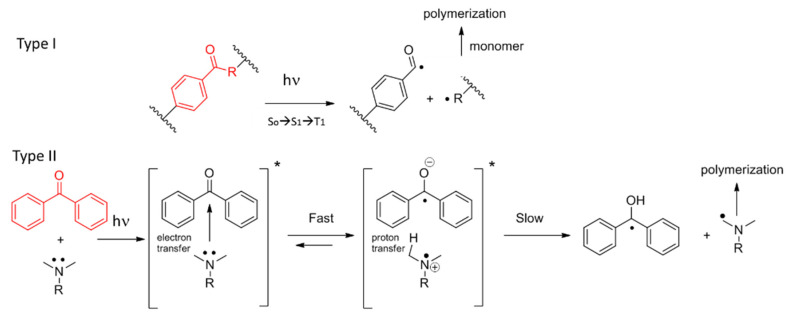
Radical generation of type I and II photoinitiators upon light irradiation.

In general, photoinduced polymerization proceeds via a chain growth mechanism, which is based on the interaction between an active center and a monomer. Therefore, the active center can be a radical, cation or anion, which is generated via an initiating step. In terms of photoinduced radical polymerization the initiation step involves a photoinitiator, generating free radicals upon UV irradiation. In respect to this, the photophysical and photochemical process of radical generation can be divided into two main mechanisms: (i) photo-scission (type I photoinitiators) and (ii) hydrogen abstraction that requires a co-initiator (type II photoinitiators) [1]. According to Figure 2, exposing a type I photoinitiator to UV light leads to homolytic cleavage processes, once the molecule has reached an excited singlet and triplet state via intersystem crossing. This cleavage commonly occurs at bonds such as C-C bonds in α position to carbonyl groups (α-cleavage), and creates two radicals via the Norrish type I reaction [1,24]. In contrast to that, the radical generation with a type II system (e.g., benzophenone/tertiary amine) is more complex and includes three steps: (i) intermolecular exciplex formation once the photoinitiator reached excited triplet state, (ii) fast electron and (iii) slow proton transfer. Notably, type II systems are less efficient because of bimolecular processes, back electron transfer and solvent cage effect in aqueous solutions [1,25,26]. In the following chapters, surface coupled type I and type II photoinitiators and corresponding co-initiators will be reviewed. Photopolymerization from coupled initiators provide—in most cases—photopolymers covalently bound to the surface. On the other hand, the immobilization of photoinitiators to the surface of (nano)particles can serve the purpose of preventing unwanted migration and leaching of photoinitiators.

### 2.1. Type I Photoinitiators 

**Figure 3:**
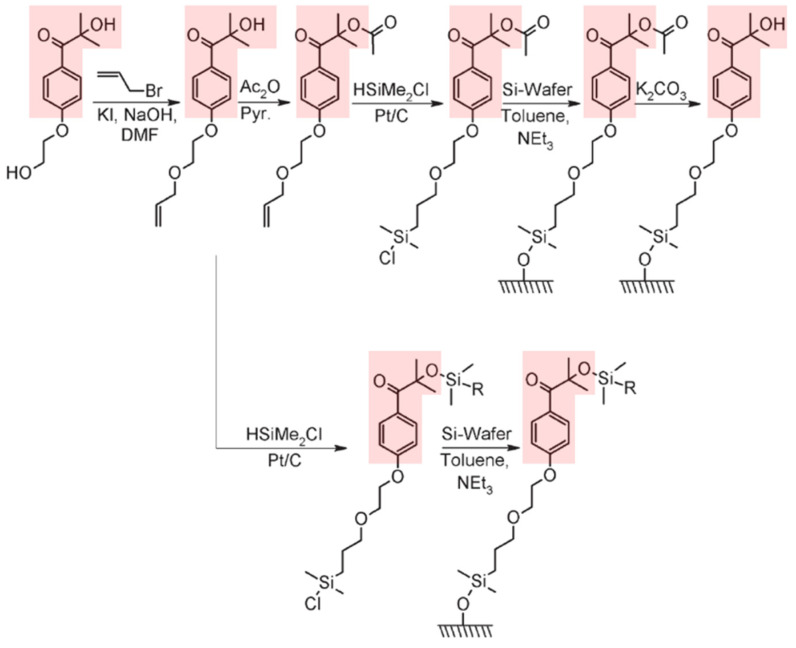
Two synthesis pathways to attach type I initiators onto silicon surfaces (slightly modified from ref. [28]; the photosensitive moiety is labeled in red).

The earliest example of a surface coupled Norrish type I photoinitiator was given by Köhler and Ohngemach in 1990. They introduced triethoxysilyl and epoxy functionalities to a (2-hydroxy-2-propyl) phenone initiator originally bearing an allylic ether or a hydroxy group in para position, employing hydrosilylation with triethoxysilane and etherification with epichlorohydrin. They then used the silane derivative as a model for the “grafting from” approach and successfully photopolymerized undiluted monomers ((N-vinyl) pyrrolidone and 2-(dimethylaminoethyl) methacrylate (DMAEMA)) from silica surfaces. However, it was not possible to polymerize styrene, (2-hydroxyethyl) methacrylate (HEMA) and (2-hydroxyethyl) acrylate (HEA) under the applied conditions. [27] Twenty years later, Rühe and coworkers showed an example of assembling a monolayer of type I initiator on silica surfaces using two different approaches starting from commercially available 2-hydroxy-4-(2-hydroxyethoxy)-2-methylpropiophenone (Irgacure 2959, see Figure 3). One approach was to protect the hydroxy group before the introduction of the silyl anchor to the molecule by converting it into a methyl ester using several steps, while the second approach was without this protection. Gradient polymer brushes of poly (methyl methacrylate) (PMMA), poly (methacrylic acid) (PMAA) and poly(dimethylacrylamide) (PDMAA) were then generated on silicon substrates employing both photoinitiator systems [28].

**Figure 4:**
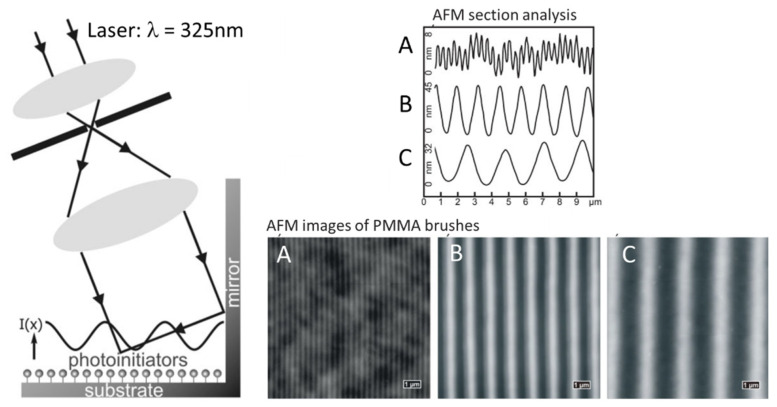
Setup for UV interference lithography and resulting polymer (PMMA) gratings (taken from reference [28]).

By using interference lithography (with a “Lloyd mirror”) the surface initiated photopolymerization yielded patterned structures. The optical arrangement (see Figure 4) included a parallel light beam, that was partly reflected from a mirror which was positioned vertically to the surface. This led to interference patterns with strong fluctuations of the light intensity irradiating the surface. Consequently, a periodical modulation of the thickness of the grafted polymer along the grating was achieved. The peak-to-valley distance was 155 nm (Figure 4A), 700 nm (Figure 4B) and 1500 nm (Figure 4C), and the thickness of the photografted polymer was in the range from 8 to 45 nm [28]. 

**Figure 5:**
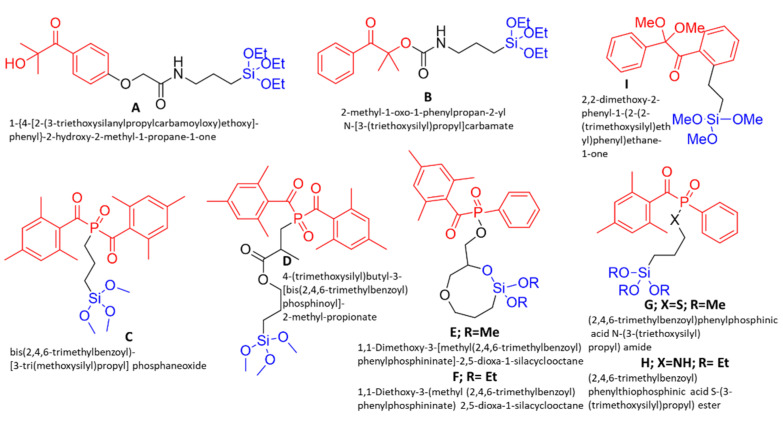
Type I photoinitiators (red) bearing a silane coupling unit (blue) for surface tethering.

Patton and colleagues used the same synthesis pathway (with hydroxy group protection) to produce silicon surfaces functionalized with Irgacure 2959. They grew a polymer brush of previously synthesized trimethylsilane-protected propargyl methacrylate which was further modified by well-known thiol-yne click chemistry after removing the trimethylsilane-moiety by immersing it into a KOH solution [29]. Moreover, they extended these orthogonal click reactions with base-catalyzed thiol-isocyanate, thiol-epoxy and thiol-bromo reactions preparing multifunctional polymer brushes [30]. More recently, Roszkowski et al., synthesized another compound bearing an Irgacure 2959 headgroup and a trialkoxysilyl unit (Figure 5A), and studied its efficiency in curing acrylic and thiol-ene resin systems. When comparing the reactivity of the functionalized initiator to the parent initiator, it was proven that the initiator bearing the silane group was very reactive and even outperformed its parent initiator. Moreover, the same initiator—coupled to silica nanopowder via well-known silanization reaction—also turned out to be efficient in polymerizing acrylic monomers and thiol-ene resins [31]. Sahin et al. investigated the influence of the amount of photoreactive groups at the surface of silica nanoparticles on the efficiency in radical mediated thiol-ene photopolymerizations. The amount of photoreactive groups was varied by the reaction conditions of the silanization procedure. It was shown that the grafting method controls size, distribution and degree of modification of the photoreactive silica nanoparticles, and therefore has an impact on the reaction kinetics [32]. 

With respect to α-hydroxyphenyl ketones such as Irgacure 2959, a hydroxypropyl radical and a benzoyl radical are formed after UV induced cleavage. The reactivity of the hydroxypropyl radical is significantly higher than that of the benzoyl radical. With regard to the so far presented photoinitiators, which were immobilized on inorganic surfaces, the less reactive radical remains immobilized on the surface. Thus, it is expected that the low molecular weight hydroxypropyl radical, which was released into the bulk, will mainly initiate photopolymerization [32]. To circumvent this, another photoinitiator with a trialkoxysilane anchor, was presented by Tan et al. in 2009. This compound was synthesized by the reaction of (3-isocyanatopropyl) triethoxysilane and 2-hydroxy-2-methyl-1-phenylpropane-1-one (Figure 5B) [33]. 

Regarding bis(acyl)phosphane oxide (BAPO) modified surfaces, the higher reactive radical remained on the surface since the phosphinoyl radical was about 1000 times more reactive than the acyl one [34]. In addition, BAPO derivatives are frequently used because they are able to form more than one radical upon UV irradiation, have an extended absorption window (λ = 360–440 nm) and show rapid photobleaching which enables deeper light penetration [35].

The first example of a BAPO based photoinitiator covalently coupled to surfaces was given by Huber et al. in 2012, who synthesized functionalized BAPO derivatives, including modification with a trimethoxy functionality (Figure 5C), which was employed to react with hydroxy groups on the surface of a cellulose material. The authors reported a slightly yellowish colored substrate which was placed in an n-hexane solution of 1H,1H,2H,2Hperfluorodecylacrylate and irradiated for 60 min, leading to a hydro- and lipophobic coating [34]. Staying with alkoxysilane functionalized BAPO, Sangermano et al. published a straight forward synthesis for 4-(trimethoxysilyl)butyl-3-[bis(2,4,6-trimethylbenzoyl)phosphinoyl]-2-methyl-propionate (Figure 5D) and successfully immobilized it on glass surfaces. Utilizing surface initiated photopolymerization they polymerized a fluorinated acrylate (1H,1H,2H,2H-perfluorooctyl acrylate) or a specifically synthesized polysiloxane to generate hydrophobic surfaces [36]. This compound was also used to grow photo-active polymer brushes bearing o-nitrobenzyl ester chromophores onto silica microparticles. Upon UV radiation, carboxylic groups were generated, which were then used to immobilize a fluorescent Alexa-546 protein [37]. The same compound was also used for the preparation of Janus II or IV hairy polymer coronas made of hydrophilic polyethylene(glycol) methacrylic ester and a hydrophobic methacrylic monomer (bearing an alkyl side chain) onto silica particles [38]. Recently, Roszkowski et al., synthesized short- and long-wavelength absorbing photoinitiators bearing trialkoxysilyl moieties, including four different BAPO-type compounds (Figure 5E–H), starting from commercially available materials. In respect to their photoreactivity, some of these initiators were compared to their parent compounds and showed comparable or even improved reactivity. However, when modifying silica nanopowder via typical silanization, it turned out that the nanopowder is a mediocre carrier for coupled photoinitiators. This is because of the low silanol content on the surface, the aggregated structure and the broad particle size distribution. Nevertheless, it was proven that photoactive nanoparticles were capable of polymerizing acrylic monomers (e.g., tetrahydrofurfuryl acrylate) and thiol-ene resins [31]. As an approach towards low extractable photoinitiators, Sahin [35] investigated silica nanoparticles bearing BAPO initiators (Figure 5D) at their surface with respect to the initiation of thiol-ene photopolymerization. Migration of the filler bound photoinitiators was examined by extraction of thiol-ene photopolymers. An elemental analysis of the extracts (phosphorus content) showed that the content of extractable photoinitiator is below detection limit when the BAPO initiator is coupled onto particle surfaces. 

Another example of a type I photoinitiator, which was attached to silica nanoparticles via silanization, was given by Moehrke et al. The silane functionalized initiator (Figure 5I) was synthesized starting from the commercially available photoinitiator 2,2-dimethoxy-2-phenylacetophenon and was used to graft n-butyl methacrylate from silica surfaces to investigate the suitability of the SP-PLP-ESR (single-pulse−pulsed-laser polymerization−electron spin resonance) technique for examination of the termination kinetics of surface-tethered macroradicals. Photolysis of this grafted initiator generated a benzoyl-type radical covalently coupled to the surface and a free dimethoxybenzyl radical. The second one is not supposed to initiate polymerization and can be further cleaved into methyl benzoate and a methyl radical which is postulated to act as a terminating species due to its high reactivity and diffusivity [39]. An example of a macromolecular photoinitiator, which includes a benzoin (type I photoinitiator) and an anhydride moiety was given by Ohar et al. This molecule is a surface modifier for filler materials, such as TiO_2_ and hydroxyapatide. Compared to the parent benzoin photoinitiator, the modified photoactive fillers showed a higher efficiency in curing resins and enhanced the surface hardness of the cured samples [40]. 

**Scheme 1:**
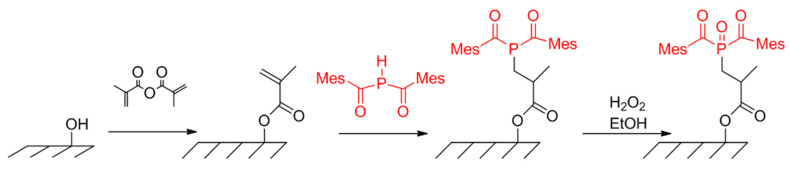
Synthetic route to attach BAPO moieties (red) onto cellulose surfaces.

Besides coupling with organosilane chemistry, Wang et al. synthesized BAPO derivatives which are capable of covalently attaching to the surface of cellulose nanocrystals (CNC). In the first step, the cellulose, bearing hydroxyl groups at the surface, was treated with methacrylic anhydride yielding surface attached methacryloyl groups. These activated surface-coupled olefins readily reacted with bis(mesitoyl)phosphane in a phospha-Michael addition in the presence of catalytic amount of triethylamine. By oxidation with aqueous hydrogen peroxide the CNC-BAPO was generated (see Scheme 1), and confirmed by elemental analysis of the yellow powder. The photoactive substrate was then used to photopolymerize MMA, butyl acrylate, N-isopropylacrylamide (NIPAM) and HEA from the surface of the CNC [41]. 

Another group of initiators for radical polymerization is based on azo initiator compounds, which can generally be activated by heat or UV radiation. The most frequently used azo initiators are 2,2-azobis(2-methylpropionitrile) (AIBN) and its derivatives, which are often used for surface modification via light induced “grafting from” processes. However, these initiators cause long curing rates due to low absorbance and long decomposition half-life. Furthermore, AIBN initiators produce two radicals by splitting off molecular nitrogen. One of the two radicals remains tethered to the surface, and the other radical diffuses into the adjacent solution starting a (unwanted) homopolymerization in the bulk [1].

**Scheme 2:**
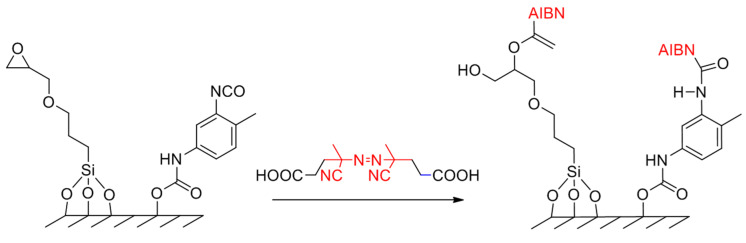
Introduction of AIBN moieties (red) onto pre-modified surfaces.

One early example of an AIBN-type initiator, which was covalently coupled to surfaces, was given by Tsubokawa et al., who polymerized vinyl monomers (MMA, styrene (St), N-vinylcarbazole) from ultrafine inorganic particles such as silica, titanium oxide and ferrite. The attachment of azo groups onto the particle surface was achieved by the reaction of epoxy groups (introduced by silanization with 3-glycidoxypropyltrimethoxysilane) with commercially available 4,4′-azobis(4-cyanopentanoic acid) (ACPA, see Scheme 2) [42]. Four years later, Tsubokawa et al. investigated the photopolymerization of azo-modified silica and titanium dioxide particles. Here, the azo groups were introduced to the surface via the reaction of ACPA with surface tethered isocyanate groups, which were generated in a previous step by the reaction between surface hydroxyl groups and toluene 2,4-diisocyanate (see Scheme 2). Compared to previous studies, the authors reported higher grafting efficiency of the photopolymerization compared to thermally induced polymerizations [43].

**Figure 6:**
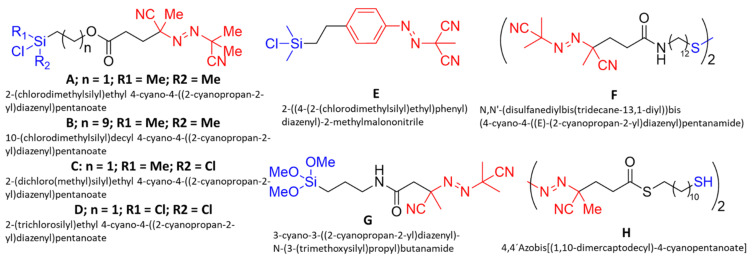
Photosensitive azo-compounds (red) with anchor moieties (blue).

In several publications, Prucker and Rühe developed multiple step syntheses for azo-compounds with a chlorosilane head group (Figure 6A–D). Moreover, these compounds contained a cleavable ester group in order to evaluate molar masses and molar mass distributions of the macromolecules (polystyrene PS, PMMA) obtained after the polymerization from silica-surfaces. Here, typical methods of polymer analysis, such as gel permeation chromatography and light scattering, were employed [44,45,46,47]. As mentioned above, AIBN-type initiators typically show rather low extinction coefficients for their n- $\pi^{*}$ transition around 350 nm. To address this, the authors synthesized another azo compound, which can be immobilized on silicon oxide surfaces, the structure of which is also shown in Figure 6E [48]. They also utilized a combination of photolithographic techniques with this “grafting from” photopolymerization method. By employing immobilized azo initiators, patterned layers of covalently attached PMMA, poly(hydroxyethyl methacrylate) (PHEMA) [49], PS as well as PS-fluorescence dye labeled methacrylate copolymer were created on silica surfaces [48].

To modify gold-surfaces, the same authors synthesized an azo-based initiator with a disulfide moiety, bearing a long alkyl group (spacer) to avoid unwanted interactions with the radicals generated by the initiator and the sulfur moiety which is used for coupling to the gold surface (Figure 6F) [48]. To circumvent free radical accelerated desorption of thiols from gold surfaces, which can hinder polymerization due to chain transfer reactions, Huang et al. utilized a crosslinked adhesion layer for initiator attachment based on mercaptopropyltrimethoxysilane. In a subsequent step they introduced azo-moieties to the surface by using a synthesized trimethoxysilane substituted azo compound (Figure 6G) [50]. Apart from these contributions, Dyer and colleagues firstly synthesized an azo initiator bearing a thiol group (Figure 6H) which was utilized for the photopolymerization of St, and mixed PMMA/PS polymer brushes from gold surfaces. Moreover, they investigated the effect of monomer concentration and reaction on the layer thickness [51,52,53]. In a cooperation with Zauscher and colleages, they presented a strategy to fabricate nano- and micropatterned polymer brush arrays made of pH- and salt-sensitive polyelectrolyte copolymers (e.g., poly(N-isopropylacrylamide-co-methacrylic acid)) using the same thiol based azo initiator [54]. 

**Scheme 3:**
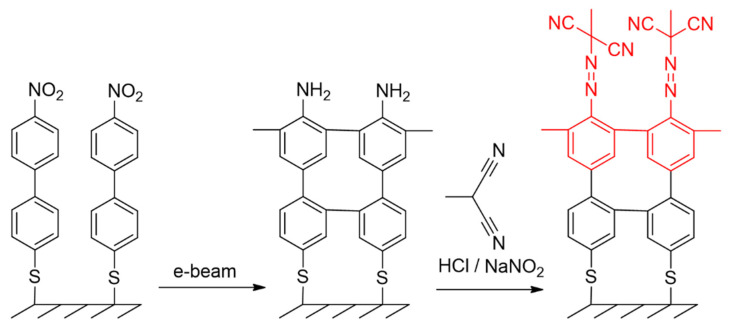
Introduction of azo moieties (red) from coupled nitrobiphenyl units using e-beam radiation.

Schmelmer et al. showed another interesting approach to couple azo groups to surfaces. In their work they used e-beam radiation to convert a self-assembled monolayer of 4′-nitro-1,1′-biphenyl-4-thiol on gold into a crosslinked 4′-amino-1,1′-biphenyl-4-thiol layer. The amino groups in terminal position were then diazotized and treated with methylmalonodinitrile to give a surface-bound monolayer of the 4′-azomethylmalonodinitrile-1,1′biphenyl-4-thiol (see Scheme 3). Irradiation with UV light generated a highly reactive phenyl radical, which remained coupled to the surface, and a relatively stable free methylmalonodinitrile radical, which is not capable of initiating radical polymerization because of its resonance stabilization. With this approach nanopatterned polymer brushes with sub-50-nm resolution and a brush height of approximately 10 nm were created [55].

### 2.2. Coupled Type II Photoinitiators

In contrast to the type I photoinitiators, type II initiators do not spontaneously generate radicals upon excitation with UV light, but readily abstract hydrogen from a co-initiator or the environment. The co-initiator may also function as an electron donor with subsequent hydrogen abstraction. Benzophenone (BP) is certainly the most popular representative of a type II photoinitiator, due to its characteristics and low cost. Other important examples of this initiator class are anthraquinone, thioxanthone (TX) and camphorquinone [24]. This chapter is dedicated to type II initiators which are covalently coupled to surfaces of different materials, starting with BP. 

#### 2.2.1. Coupled Benzophenone (BP)

**Scheme 4:**
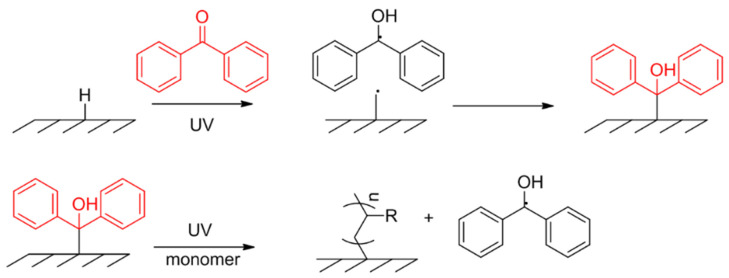
Sequential method for photografting polymers using benzophenone (BP) as surface coupled photoinitiator (red). BP coupling comprises two steps: (i) UV triggered hydrogen abstraction with subsequent recombination, and(ii) re-initiation by UV light in the presence of monomers leading to grafted polymer chains.

The earliest example for coupled BP initiators was given by Yang and Ranby in 1996 who suggested a living nature of the photopolymerization based on BP chemistry. A solution of BP and methacrylic acid (MAA) was placed between two low-density-polyethylene (LDPE) films using a micro syringe. As a consequence of irradiation for 1 min, BP abstracted hydrogen from the LDPE surface, generating reactive carbon centered radicals which initiated polymerization of MAA. In their earlier studies they found that termination reactions are mainly carried out by combination of the growing chain and the semipinacol radical. The same procedure was done with different monomers (St, MMA) leading to different end-capped polymers. Upon a second UV irradiation it was found that all these end groups, except for the one consisting of acrylic acid (AA) and benzophenone, could be reinitiated for polymerization of methacrylic acid [56]. A disadvantage of this method is the significant formation of homopolymers in the solution since BP also abstracts hydrogen from monomers (and solvent molecules). To circumvent this, Ma et al. suggested a two-step method (Scheme 4), in which the substrate was, firstly, immersed in a saturated solution of benzophenone, then washed and dried and subsequently irradiated with UV light. It was supposed that BP abstracts hydrogen from the substrate which then immediately recombines with the semipinacol radical in the absence of monomers in solution. In the second step the monomer solution was directly cast onto the substrate and exposed to UV light, reinitiating and starting the polymerization. With this sequential method, the authors grew PMAA and poly (ethylene glycol) mono methacrylate onto hydrophobic porous polypropylene (PP) membranes and reported a four times larger amount of grafted polymer in comparison to the method of Yang and Ranby [57]. Later they extended this method for application on different substrates (cellulose acetate, poly (vinylidene fluoride)). Furthermore, and for the first time, they provided direct evidence for the covalent coupling of the grafted polymer using GPC. For this, they dissolved low molecular weight PS and BP in benzene and exposed a thin film of this solution to UV light, resulting into BP that is coupled to PS. After washing with ethanol to remove unreacted BP, methacrylic monomers were photopolymerized onto BP-modified PS macromolecules in solution. A comparative GPC-study between modified and virgin PS, revealed a higher molar mass of the modified PS and therefore evidenced covalent coupling [58]. 

Several groups then used this two-step “photo-grafting” method with surfaces which provide hydrogen for abstraction (for example polyethylene terephthalate, polylactic acid, etc.) [59,60,61,62,63]. Castell et al. used BP and numerous BP derivatives to modify surface properties, more specifically the wettability and adhesion, of chemically inert PP films without subsequent polymerizations and performed a series of experiments to evaluate impact factors on the grafting reaction. Depending on the structure of the utilized BP derivative, the author reported an increase of the surface energy from 26 (pure PP) up to 36 mN/m and pointed out that oxygen might inhibit the photografting reaction. In a subsequent work, surface wettability and adhesion were further improved by photografting MAA, pentaerythritol triacrylate, and 2-hydroxyethyl acrylate from BP derivatized PP surfaces [64,65]. The two-step method, proposed by Ma et al., was also used for another type II initiator, namely, 1-hydroxycyclohexyl phenyl ketone, to increase the wettability of the negative tone photoresist SU-8, through photopolymerization of tetraethyleneglycol dimethacrylate and HEMA [66].

**Figure 7:**
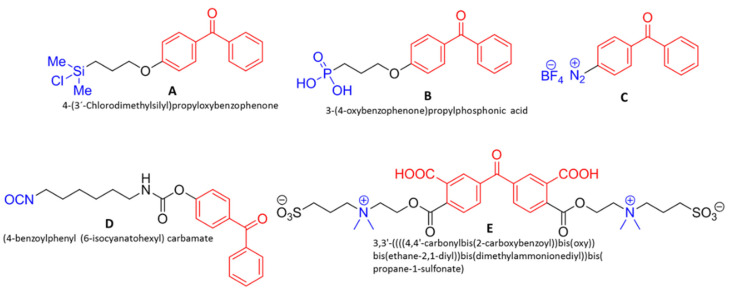
BP derivatives with different coupling units (photosensitive groups are labeled in red; anchor moieties are labeled in blue).

As already described, the above-mentioned method is limited to surfaces, which provide hydrogen for abstraction in the course of the photo-grafting process. In contrast, the following section deals with non-hydrogen containing surfaces, which are modified with BP utilizing different coupling chemistries. Starting with well-known silanization, Prucker et al., developed a simple and high yield synthesis of a BP derivative bearing a silyl anchor to typically attach to oxidized surfaces such as silica (Figure 7A). Performing a “grafting to” approach they successfully grafted PS and poly(ethyloxazoline) (PEOX) onto SiO_2_ surfaces by illumination of the polymer layer which had been spin-cast onto the surface modified with BP [67]. Based on this fundamental work, Kado et al. showed an impressive photolithographic pattering method using a combination of a photo-cross-linkable polymer bearing an acrylic functionality, which was sequentially deposited by the Langmuir–Blodgett method onto photoreactive BP-silica surfaces. The self-crosslinking and the photo-grafting upon UV radiation lead to patterned multilayer polymer nanosheets which displayed interference coloring corresponding to different film thicknesses [68]. One further example of an application was recently given by Braun et al., who functionalized indium tin oxide coated glass substrates with the same silane bearing BP-derivative to assemble liquid crystal test cells. The test cells were filled with reactive mixtures of acrylate monomers and exposed to UV light in order to generate a surface grafted polymer network with enhanced electro-optic responses [69]. BP and its derivatives also found application in biochemistry because they can be manipulated by ambient light and activated by light in the range of 345–360 nm, avoiding protein damage at this region. Moreover, they react with comparatively inert C–H bonds in a broad range of different chemical environments and are more stable than diazo esters, aryl azides and diazirines. Therefore, the silane derivative of BP was used to immobilize biomolecules onto a fiberoptic silica surface [70], to develop a polymer-tethered phospholipid bilayer consisting of a BP-derivatized glass surface, a polymer layer and a tethered phospholipid bilayer [71], and to create a broad range of polymeric coatings for biomedical applications [72]. 

Staying with biochemical applications, Griep-Raming et al. used a phosphonic acid anchor to attach BP moieties onto the surface of titanium. In comparison to organosilanes, the hydrolytic stability of phosphonic acid monolayers on titanium is higher, which is important for long lasting implants. The BP-phosphonic acid compound (Figure 7B) was synthesized in a three-step synthesis in very good yields and was coupled to the surface by convenient depositing-heating-washing cycles. The authors then reported successful grafting of PHEMA and PS and pointed out that phosphonic acid anchors can also be used to couple the initiator to tantalum, aluminum, mica, zirconium, silicon, steel, stainless steel, copper, brass, etc. [73].

A slightly different coupling approach was shown by Raghuraman et al., who used a hydro silane for the attachment onto SiO_2_ surfaces. Compared to chlorosilanes the hydrosilane does not need strict exclusion of moisture during self-assembly but can be applied under ambient conditions. With this, PS was grafted to the surface using the BP route and also an ATRP initiator (see Section 3.2) was introduced with the same approach to the surface, followed with surface-initiated polymerization of *tert*-butyl acrylate [74].

Leaving silanization chemistry, Gam-Derouich et al. showed the covalent coupling through electroreduction of the synthesized diazonium salt BP derivative (Figure 7C) to different surfaces. The authors initiated photopolymerization of model monomers, namely, St, MMA and HEMA from gold-coated silicon substrates, which were previously electro-grafted with the BP diazonium salt (BP-DS) [75]. In their following studies they grafted a superhydrophilic polymer onto BP-DS functionalized stainless steel and gold surfaces using surface-initiated photopolymerization for biomedical applications [76], and extended this “grafting from” method to different substrates (glassy carbon, indium tin oxide) and monomers (MAA, poly (4-vinyl pyridine)), also using dimethyl aniline as co-initiator [77]. Another impressive application was given by the same group, developing molecularly imprinted polymer grafts on gold or gold nanoparticles for the specific and selective detection of dopamine [78] and melamine [79]. 

**Scheme 5:**
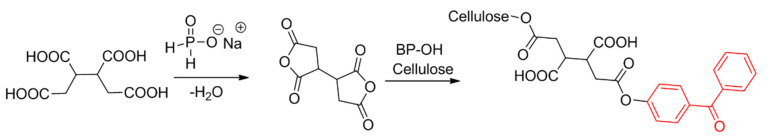
Introduction of a benzophenone unit (red) to cellulose.

Surface modification of cellulosic materials utilizing BP was demonstrated by Hong et al. The authors used butane tetracarboxylic acid which was reacted with sodium hydrophosphite resulting in the corresponding dianhydride. This bifunctional linker reacts with the hydroxy groups of the cellulosic material and the hydroxy group of 4-hydroxybenzophenone simultaneously (see Scheme 5). Upon UV irradiation the BP moiety abstracts hydrogen from the cellulosic material, thus generating two radicals which are both capable of photopolymerizing acrylamide (AAm). The so-prepared cotton fabrics showed enhanced thermal stability and, through a chlorination process, excellent anti-bacterial properties were obtained [80]. 

Another approach towards modified cellulose nanocrystals was recently shown by Biyani et al. They synthesized an isocyanate bearing BP derivative (Figure 7D) which was reacted with cellulosic hydroxyl groups at elevated temperatures in the presence of a catalytic amount of dibutyltin dilaurate, forming a urethane group. The cellulose nanoparticles were incorporated into a polymer (ethylene oxide/epichlorohydrin copolymer) forming covalent bonds with the macromolecules of the matrix upon UV radiation. This led to a stiffness increase as well as reduced swelling and softening upon exposure to water [81]. 

Photoinitiator-modified montmorillonite was recently reported by Melinte et al., who synthesized a novel initiator bearing a BP core and a quaternary ammonium unit, which was capable of coupling to cloisite layers via a cationic exchange of Na^+^ (Figure 7E). Furthermore, acrylic monomers (urethane dimethacrylates) were photo-crosslinked with modified montmorillonite as the initiating species without the use of solvents, leading to a hybrid nanocomposite coating [82].

#### 2.2.2. Coupled Eosin

Furthermore, eosin is a well-known type II photoinitiator. This dye strongly absorbs light (ε = 60,800 l M^−1^ cm^−1^ at 540 nm), and is then transferred into its singlet state followed by a rapid intersystem crossing (ISC) to the lowest energy triplet state, with a lifetime of 24 µs. Electron transfer from the co-initiator (commonly tertiary amines) leads to a radical eosin anion and an amine radical cation, followed by a proton transfer generating an eosin radical and a more reactive α-amino radical which is expected to start the polymerization. In terms of surface coupled eosin initiator, the subsequent photopolymerization is meant to be surface mediated since the reactive amino radical is generated in the vicinity of eosin, which is surface-tethered. Eosin is, because of visible light absorption, highly suitable for applications which require a high penetration depth and in which a high dose of UV light would destroy proteins or DNA. Furthermore, the use of visible light avoids unwanted bulk polymerization, which is often observed for irradiation with UV light [83,84,85,86].

**Scheme 6:**
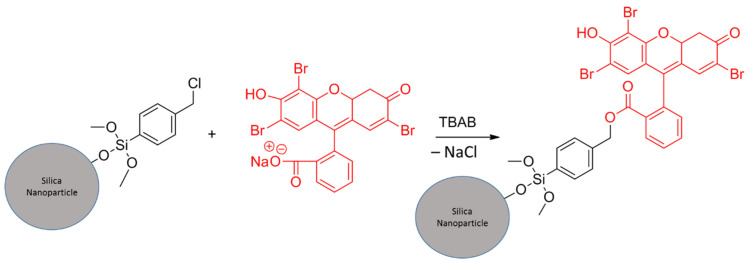
Synthesis pathway towards silica nanoparticles modified with eosin (red). A phase transfer catalyst (tetrabutylammonium bromide; TBAB) was used.

Satoh et al. reported eosin modified silica nanoparticles via the reaction between sodium carboxylate groups of eosin Y and benzyl chloride groups in the presence of a phase transfer catalyst (see Scheme 6). Subsequent photopolymerization of St, MMA, AA and acrylonitrile was carried out in the presence of oxygen and ascorbic acid as a reducing agent, and determined the wettability of the grafted silica nanoparticles [87].

**Scheme 7:**
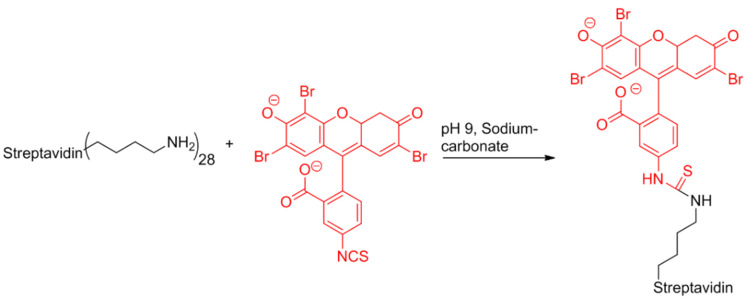
Synthesis of photo initiating eosin (red) species bearing a streptavidin anchor.

Hansen and co-workers coupled eosin, which was functionalized with a streptavidin protein unit according to Scheme 7, to biochip glass surfaces, selectively carrying biotinylated oligonucleotides, through a biotin-streptavidin bond. Upon irradiation with visible light and being in contact with a monomer (polyethylene glycol (PEG) -diacrylate) containing a co-initiator, a hydrogel was exclusively grown at spots, where the photoinitiator was allocated. This served as a visible-light-polymerization-based-amplification for visual biotin detection. For this purpose, eosin-5-isothiocyanate was reacted with amino groups of streptavidin leading into the formation of a thiourea bond [86]. Streptavidin-eosin was further used to encapsulate mammalian cells by photopolymerization of hydrogel precursors from the cell surface in order to manipulate transport processes at the surface [88]. The same authors also showed that PEG coated cells are protected in harsh environments, for example, during antigen specific lysis, and proved a strong correlation between the density of surface immobilized streptavidin-eosin and the yield of intact cells during antigen specific lysis [89].

Cho et al., exploited the reaction between polyamines (polyethylenimine, spermine, polypeptide) and eosin-5-isothiocyanate to create eosin-bearing macromolecules, which build a polyplex with plasmid DNA. After surface initiated photopolymerization with water-degradable acrylic monomers an acid-degradable polyketal shell with the gene-carrying polyamine-core was received. The polyketal shell of this gene-delivery system was degraded in certain pH-defined environments exposing the DNA core to the targeted cell [90]. 

Surface modification of cotton fabrics using eosin as a covalently bound photoinitiator was investigated by Stenava et al. The authors synthesized an eosin derivative bearing a pyridinium salt unit, which was added to the dye bath. Surface initiated photopolymerization of 4-propylamino-N-ethylmethacrylate-1,8-naphthalimide (synthesized by the authors) and N,N-methylenebisacrylamide in the presence of a co-initiator yielded a fluorescent and pH-responsive hydrogel layer [91]. 

Kizilel et al. used amino-functionalized glass or silicon substrates, modified by silanization with 3-aminopropyltriethoxysilane APTES to introduce eosin moieties to the corresponding surface. Therefore, the carboxylic group of the eosin-molecule was reacted with Woodwards Reagent K (WRK), and subsequently formed an amide bond with the amino-functionalized substrate. Photopolymerization of PEG-diacrylate and 1-vinyl-2-pyrrolidinone was carried out under mild conditions in the presence of triethanolamine, generating patterned hydrogels, which have tissue-like physical and mechanical properties [85]. In a subsequent contribution the same authors exploited the reaction between pendant amino groups and the carboxylic acid group of eosin (activated with WRK) to generate hydrogel multilayers using PEG amino acrylate along with other hydrogel precursors through sequential introduction of eosin Y and photopolymerization [92]. 

#### 2.2.3. Coupled Thioxanthone

Thioxanthone (TX) is another widely used type II photoinitiator. The research goes back to 1981. Upon irradiation with UV light, TX reaches its triplet state via intersystem crossing. From its triplet state TX reacts with hydrogen donors, such as amines, forming a co-initiator radical and a TXH. ketyl-radical. The latter can dimerize, carry out disproportion reactions, terminate radical polymerization in some cases and react with molecular oxygen. A deeper look into TX photophysics and photochemistry can be obtained from the literature (ref. [93]). 

**Scheme 8:**
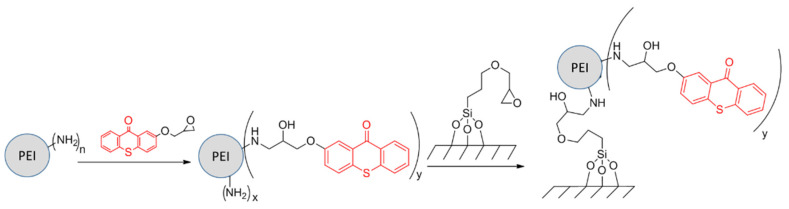
Synthesis of the macroinitiator HPTX, and subsequent surface coupling (photosensitive TX-moieties are labeled in red).

Water-compatible photoinitiators derived from thioxanthone are widely used in printing inks, coatings, microelectronics and photoresists. For this purpose, Jiang and coworkers synthesized a hyperbranched TX derived macrophotoinitiator (HPTX, depicted in Scheme 8) using dendritic poly(propyleneimine) (PEI) which is a well-defined and highly branched molecule with a high density of amino groups. PEI is composed of tertiary amines in the core and of secondary as well as primary amines in the periphery, which can be easily utilized for the reaction with epoxides such as epoxy derivatives of TX. The resulting HPTX includes photo-initiating species as well as the co-initiator moiety in one molecule and shows photobleaching properties [94,95]. In a further contribution of the same group, HPTX was covalently attached to silica surfaces via nucleophilic addition reaction between surface tethered epoxy groups and amino groups of the initiator leading to a self-assembled monolayer. The epoxy groups at the silica surface were preliminary introduced via silanization with 3-glycidyloxypropyl trimethoxysilane. Through surface initiated photopolymerization of NIPAM, a coating with temperature responsive properties was gained [96]. Exploiting the photobleaching properties of HPTX, patterned binary polymer brushes were produced. HPTX was immobilized onto silica surfaces by the above-mentioned procedure. In the absence of monomers, the samples were exposed to UV light through a photomask in order to photobleach and therefore deactivate the initiator within a defined area. Subsequent photopolymerization of MMA created polymer brushes coupled to the non-bleached area. Binary brushes were then developed using a solution of St and free TX to grow PS-brushes from the formerly bleached area, taking into account the remaining immobilized amino groups, which can still act as co-initiators for polymerization [97]. Chen and co-workers extended the scope of this technology to silica particles. TX derivatives are suitable for this application because of their excellent UV-absorbance in the spectral region 365–400 nm, which is not affected by the shield of light caused by particles. The introduction of HPTX was, in a similar way to the above-described method, based on the reaction between epoxy and amino functionalities. After surface initiated photopolymerization of MMA, the obtained particles were incorporated in a PMMA matrix to enhance thermal and mechanical properties [98]. 

**Scheme 9:**
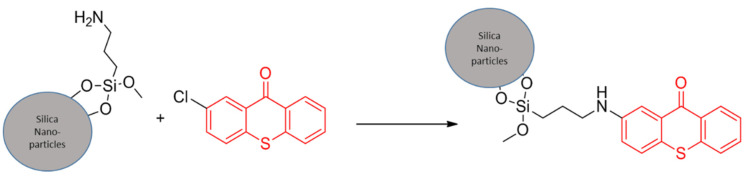
Introduction of TX (red) onto silica nanoparticle surfaces.

Recently, Li et al. introduced the TX moiety onto the surface of silica nanoparticles. Nanoparticles were firstly modified with APTES via silanization leading to surface anchored amino groups. These groups were converted with a TX derivative bearing a chlorine substituent, developing a covalent link between TX and silica nanoparticles as shown in Scheme 9. In a comparative study, these modified nanoparticles exhibited higher efficiency in photopolymerizations than the parent TX initiator and prominently showed good dispersibility in epoxy acrylate resins. Furthermore, cured samples with these modified nanoparticles had a significantly lower amount of extractable residual photoinitiator which offered advantages with respect to environmental issues [99]. 

**Scheme 10:**
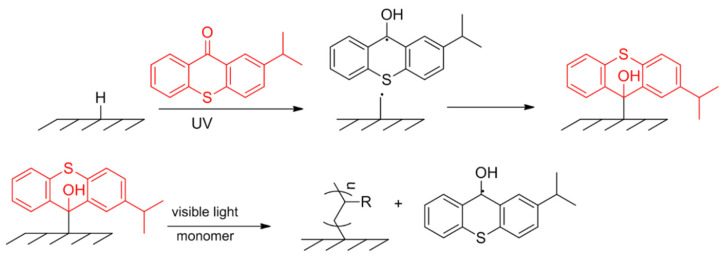
Two step method for thioxanthone immobilization and subsequent grafting-from reaction (photosensitive moieties are labeled in red).

As was done with BP, a derivative of TX, namely, isopropyl-TX (ITX), was also coupled to polymer surfaces by Bai et al., using the two-stew method. UV illumination of ITX between two LDPE films leads to surface tethered isopropyl thioxanthone-semipinacol through excitation, subsequent hydrogen abstraction and recombination (see Scheme 10). 

The resulting “dormant” group exhibits light-absorption in the visible light region, and was employed to graft glycidyl methacrylate (GMA) from the LDPE-surface via visible light induced photopolymerization. The authors also pointed out the “living” characteristics of this grafting polymerization since the thickness of the grafted layer was controlled by altering the irradiation time (linear increase of layer thickness versus irradiation time) [100]. This technology found application in biomedicine where the “dormant” groups at the surface of LDPE were used to generate polymer brushes of poly(ethylene glycol) methyl methacrylate for anti-fouling properties. Furthermore, dormant ITX moieties at the end of the polymer-brushes were again utilized for a crosslinking copolymerization of GMA and polyethylene glycol diacrylate (PEGDA), generating a layer which can bind proteins via epoxy-amine reactions onto the resulting microarray. Both photopolymerizations were carried out under visible light [101]. A technique which combines photolithography and visible light induced photopolymerization was shown by Zhao and co-workers. After coupling ITX onto LDPE surfaces, using the same procedure, PEGDA was grafted from the surface in a patterned manner using a photomask. To verify the living nature of this grafting polymerization, a second re-initiating illumination step was performed with a solution of PEGDA and sodium acrylate resulting in a 3D-patterned surface. Furthermore, a reactive two-layer 3D pattern of poly (acrylic acid) (PAA) and PEGDA with a morphology of lateral stripe on vertical stripe (for example) was prepared and employed to separately immobilize model biomolecules [102].

**Scheme 11:**
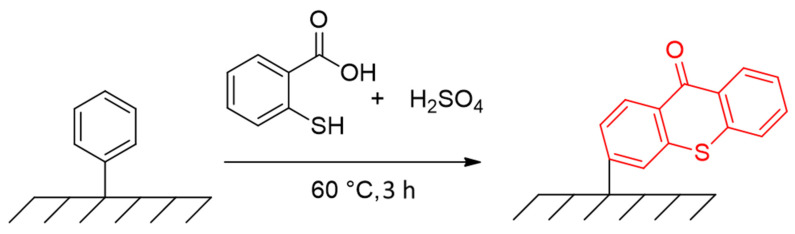
Introduction of the TX moiety (red) onto PS surfaces.

In an unconventional approach by Fukumori et al., the TX photoinitiator was directly formed on the surface of solid polystyrene. In this reaction thioxanthone was readily formed on the surface by utilizing phenyl groups of PS in the presence of sulfuric acid and thiosalicylic acid (see Scheme 11). Surface initiated photopolymerization of poly(N-isopropylacrylamide) under visible light led to temperature-responsive cell culture surfaces [103].

#### 2.2.4. Coupled Antraquinone

Another type II initiator, namely, anthraquinone, was also used for surface modification, but to a lesser extent when compared to above mentioned BP, eosin and TX. Liu et al. used water-soluble anthraquinone-2-sulfonate sodium as an acid dye to modify polyamide fibers via a simple dyeing process, which leads to ionic bond formation. Upon UV irradiation they grafted AA onto the fibers [104]. The same group produced self-cleaning cotton-fabrics with antimicrobial functions against both Gram negative and Gram-positive bacteria, using 2-anthraquinone carboxylic acid. This initiator was attached to the surface through a N,N-carbonyldiimidazole catalyzed esterification between the hydroxy groups of the cellulose material and the carboxyl groups of the initiator [105]. 

### 2.3. Surface Immobilization of Synergists for Benzophenone and Thioxanthone

Another approach to use BP as a photoinitiator for surface-initiated polymerization is to immobilize the corresponding co-initiator (hydrogen donor). Commonly used co-initiators for BP are tertiary amines which form an exciplex with BP upon UV radiation. Immobilizing the synergist seems to be favorable, since the amino-substituted alkyl radical is highly reactive in initiating polymerizations [106]. 

**Scheme 12:**
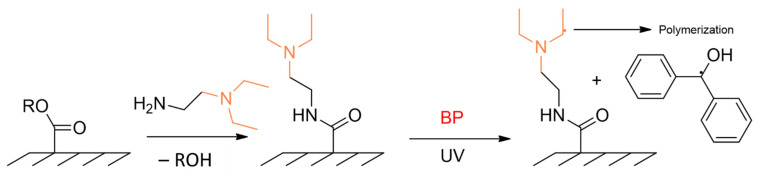
The immobilization of amine synergists onto surfaces and subsequent type II photopolymerization (the co-initiating species is labeled in orange).

He et al. extensively studied BP synergist immobilization for the modification of polymeric membranes with different pore sizes. In their first publication in 2006 the tertiary amine was introduced through an aminolysis reaction between ester groups on the surface of hydrophilized PP and etched poly (ethylene terephthalate) (PET) membranes for microfiltration, and N,N-diethylethylenediamine according to Scheme 12. This coupling method was supposed to be possible for all polymers, which bear ester groups at their surface. With BP in solution the authors grafted a water-swellable layer of poly (acrylamide) (PAAm) from the PET and PP surfaces and analyzed the grafting mechanism under variation of the irradiation time, monomer and BP concentration, as well as the synergist content on the surface. Intense UV irradiation accelerated the grafting process but led to a loss of surface selectivity. Furthermore, for ideal surface selectivity a low concentration of photoinitiator was required. The authors also pointed out that a potential competition between the monomer, which serves as a solvent, and the membrane could decrease the degree of grafting and the surface selectivity [106,107]. Using this well-studied synergist immobilization method, the authors prepared anion-exchange membranes with different architectures of grafted layers [108], and a protein-selective copolymer layer on PET track-etched membranes [109]. 

**Figure 8:**
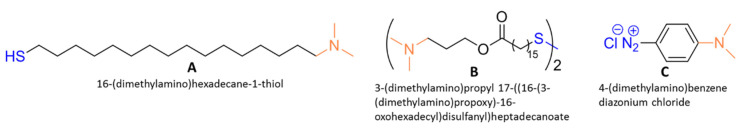
Examples for synergists bearing dimethylamine units (orange) and different coupling groups (blue).

Dyer et al., designed a series of dimethylamino terminated thiols (Figure 8A,B) and disulfides to graft polymer brushes from gold surfaces. When grafting PS and PMMA from an amino functionalized Au surface, the authors surprisingly obtained a higher grafting degree in the absence of BP. They also compared an azo-initiator with this tertiary amine system, and reported much thicker films in about half the irradiation time in cases where the tertiary amine system is used [110]. 

To prepare bioactive polymer grafts, Gam-Derourich et al. electroreduced N,N-dimethylamino benzenediazonium chloride (Figure 8C) to provide a 4-(dimethylamino)phenyl hydrogen donor layer on gold under nitrogen abstraction. Using free BP and HEMA in solution the group reported densely packed PHEMA layers which completely covered the gold substrate [111]. 

According to the study of Kim et al., BP is also able to abstract hydrogen from OH-groups at silica nanoparticle surfaces upon UV irradiation. The resulting oxygen radical initiated chain growth of MMA, leading into a covalent Si-O-C bonding as it was proven by FT-Raman analysis. The modified silica nanoparticles contained 31% of PMMA by weight and therefore showed improved monodispersity without agglomeration [112].

**Scheme 13:**
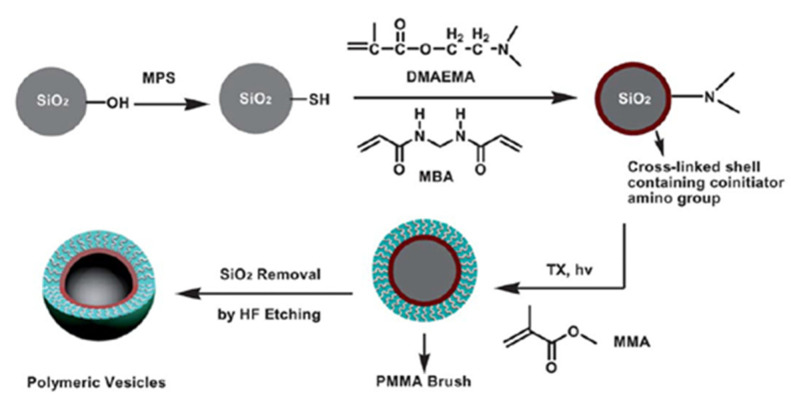
Production of polymeric vesicles using a combination of conventional polymerization and subsequent grafting from photopolymerization utilizing thioxanthone photochemistry (taken from ref. [113]).

Synergist immobilization was also examined by Chen et al. for TX creating polymer vesicles with well-defined PMMA brushes onto silica nanoparticles. The modification was done using (3-mercaptopropyl) trimethoxysilane in order to introduce thiols onto the surface of synthesized silica nanoparticles (see Scheme 13). A crosslinked shell was then coupled onto the surface via free radical polymerization of 2-(dimethylamino)ethyl methacrylate, which bears the tertiary amine group, and N,N-methylenebis(acrylamide) as the crosslinking agent. The polymerization was carried out in solution at elevated temperatures using AIBN as a radical initiator. In this process the surface tethered thiol groups were expected to undergo transfer reactions with the propagating chains, therefore leading into the polymeric shell. Subsequent surface initiated photopolymerization of MMA, using TX in solution and the coupled amino group as the co-initiator, followed by removing the SiO_2_ core with aqueous HF, yielded polymeric vesicles [113].

To provide a quick overview of Section 2, a table (Table 1) summarizing surface coupled type I and II photoinitiators and co-initiators (synergist) will be given. In the first column the chemical structure of the corresponding compound is described. Thereby, the wavy line suggests the substrate surface to which the (co)-initiator is coupled. The second and third columns include the modified material and the corresponding reference.

## 3. Light Mediated Controlled Radical Polymerization from Surfaces

Among different controlled and “living” polymerization techniques, radical-based strategies are frequently used because of their compatibility with both aqueous and organic media as well as their high tolerance towards a wide range of functional groups. Controlled polymerization techniques for polymer brush synthesis include (i) surface-initiated reversible addition fragmentation chain transfer (SI-RAFT) polymerization, (ii) surface-initiated photoiniferter-mediated polymerization (SI-PIMP), (iii) surface-initiated atom transfer radical polymerization (SI-ATRP) and (iv) surface-initiated nitroxide-mediated polymerization (SI-NMP), whereas the latter is commonly conducted at high temperatures [21] and will not be further discussed in this contribution [4,20,21]. In terms of surface initiated photopolymerization the SI-PIMP concept will be discussed in detail since the photo-initiating species is directly coupled to different surfaces in the given examples below. In addition, some examples of surface initiated photopolymerizations based on the ATRP and the RAFT technique, in which the photo initiating species, or better to say the photoredox catalyst, is not coupled to surfaces, will also be presented.

### 3.1. Surface-Initiated Photoiniferter-Mediated Polymerization (SI-PIMP)

**Figure 9:**
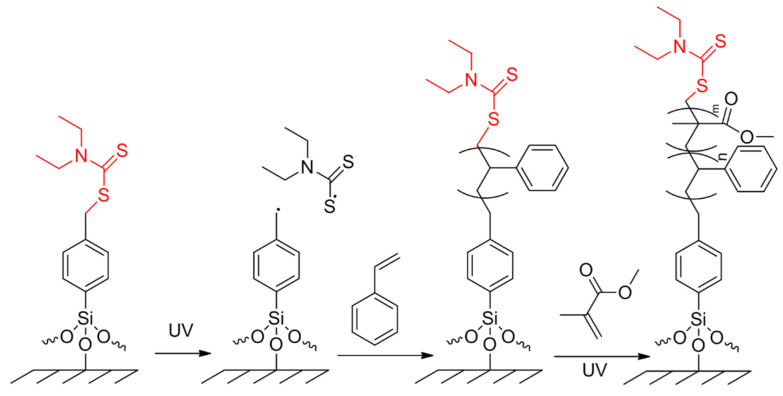
Exemplary preparation of copolymer brushes by SI-PIMP from a benzyl-N,N-diethyldithiocarbamate derivatized substrate (photosensitive group is labeled in red) [4,114].

The concept of polymerization methods, which are mediated by photoiniferters was pioneered by Otsu et al. in 1982 [114]. Iniferters are molecules, which induce radical polymerization and simultaneously act as chain transfer and chain terminating agents. The controlled nature of the polymerization is based on the photolytic cleavage of the iniferter-molecule leading to a carbon-centered radical, which readily undergoes addition reactions with monomers initiating the chain propagation, and a persistent dithiocarbamyl radical. Herein the dithiocarbamyl radical does not initiate the polymerization but acts as a chain transfer agent and also induces reversible termination, yielding polymers with reactive chain ends, which can be reinitiated to form copolymers [4]. The mechanism of SI-PIMP, including photolytic cleavage of the iniferter molecule and subsequent “grafting from” (co)-polymerization of acrylic or vinyl monomers is summarized in Figure 9. Within this example, the iniferter molecule is coupled to an appropriate surface via silanization chemistry as an example. However, more detailed information about the iniferter concept, different iniferter-molecules and living radical polymerization, can be found in ref. [115] and ref. [116]. 

The SI-PIMP based “grafting from” approach is a widely used method to prepare numerous polymer brushes and coatings on different surfaces, [21] and provides the possibility to adjust chemical compositions, film thickness and grafting densities of the produced layers by regulating the monomer concentration and the UV exposure time. Moreover, it does not require any toxic catalysts making this technology suitable for biomedical applications [117,118].

**Scheme 14:**
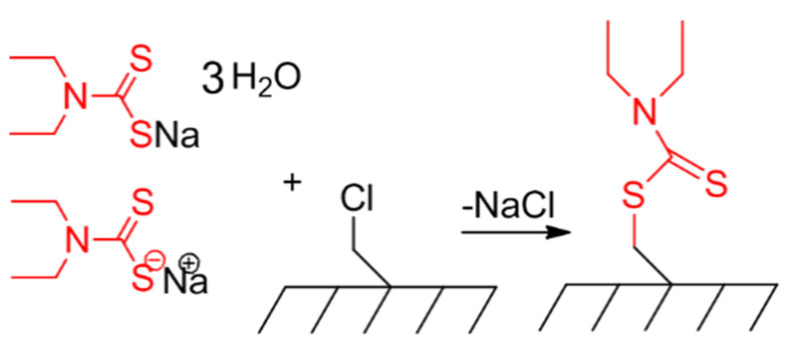
Coupling strategy to introduce a photoiniferter functionality (red) to chloromethylated surfaces using sodium N,N-diethyldithiocarbamate.

Otsu et al. also reported the first example of SI-PIMP by coupling a N,N-diethyldithiocarbamat derivative onto PS surfaces via hydrolysable ester-bonds. The authors used polystyrene-divinylbenzene- copolymer beads, which were chloromethylated and subsequently reacted with N,N-diethyldithiocarbamyl acetate yielding a photoreactive solid PS (see Scheme 14). As a proof of concept, the authors grafted PMMA and PS from the modified surface to synthesize PMMA-PS-block copolymer brushes [119].

Matsuda and colleagues extended this concept using a PS film which was crosslinked by γ-rays from a ^60^Co source in a preliminary step and subsequently immersed into a solution of chloromethylethylether and ZnCl_2_. The chloromethylated PS-film was kept in a solution of sodium- N,N diethyldithiocarbamate trihydrate (DC-H) to immobilize the photolabile group onto the PS surface. Precise control of the molecular architectures of PDMAA, poly- N-[3-(dimethylamino)propyl]acrylamide, PMAA and PS under UV radiation at room temperature was reported [120]. The same group later presented a two step-method for highly spatio-resolved graft-copolymerized surfaces, in which the stem and the branch design were comparatively well controlled using the photoiniferter concept. In an initial step they synthesized a photoactive glass surface using a well-known silanization approach with chloromethylphenylethylsilane. The chloromethylated glass was then converted into dithiocarbamate (DC) derivated glass using DC-H. After photopolymerization of chlorovinylstyrene, a second functionalization with DC-H was done leading to polymer chains with DC-derivatized side-groups from which MAA, N,N-dimethylacrylamide (DMAAM), sodium methacrylate and poly(ethylene glycol) methacrylate (PEGMA) were photopolymerized [121]. In a further publication, Matsuda et al. used the same coupling strategy (chloromethylation and subsequent treatment with DC-H, Scheme 14) to develop DC-derivatized polyurethane surfaces, from which poly(PEGMA) and PDMAA were photografted in order to achieve highly water-wettable surfaces [122]. The nucleophilic substitution of surface tethered chlorine units, which were covalently coupled to surfaces by commercially available DC-H via a SN_2_ mechanism, [123] was further exploited by many groups modifying cellulose membranes with MAA and ethylene glycol dimethacrylate [124], inert cycloolefin polymer substrates with an upper PAA and a bottom poly (PEGMA) layer [125] and different silica microparticles with PMMA [123]. Ma et al. prepared iniferter functionalized magnetic microspheres which consisted of Fe_3_O_4_. An SiO_2_ layer prepared from tetraethyl orthosilicate improved the microsphere stability and provided the anchoring site for initiator-attachment using p-chloromethyl, phenyltrimethoxysilane and DC-H. After photopolymerization the resulting polymer brush showed antifouling and antibody immobilization properties [126]. A slightly different approach was shown by Ulbricht and colleagues, who modified hydrophilized PP microfiltration membranes by utilizing three straightforward solid phase reactions. First, aminolysis was executed to introduce amino groups onto the surface, followed by a reaction with 2-bromoisobutyryl bromide introducing a bromine leaving group at the surface which was finally reacted with DC-H leading to an iniferter-modified PP-surface from which a copolymer of AAm and ethylene glycol dimethacrylate were polymerized [127].

**Figure 10:**
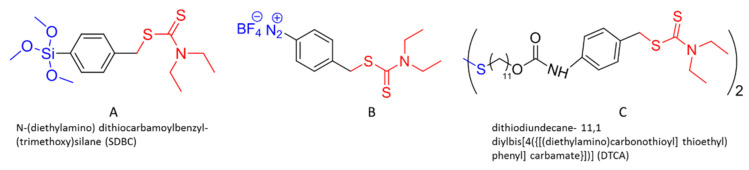
Iniferter compounds (red) bearing different anchor moieties (blue).

In the early 2000s, De Boer et al. synthesized an organosilane containing a DC group (Figure 10A, SDBC). In this fundamental work, they modified silicon-wafers by graft-photopolymerization of MMA and St [128]. SDBC was then used in several publications. Rahane et al. used SDBC to modify silicon surfaces and studied the kinetics of SI-PIMP of MMA by measuring the layer thickness as a function of reaction-time, monomer concentration and light intensity via variable angle ellipsometry. The results indicated a pseudo-living nature of the photopolymerization due to irreversible bimolecular termination reactions, which decreased the amount of surface free radicals with increasing exposure time [129]. In later publications, Rahane et al. proposed a kinetic model for SI-PIMP [130], and suggested using tetraethylthiuram disulfide to reduce irreversible termination reactions and to provide controlled radical polymerization behavior [131]. Schönherr and colleagues used SDBC to grow thin films of PAAm and covalently crosslinked, swellable hydrogel brushes from iniferter-functionalized silicon substrates, and reported thick films (up to 1 µm) within 1 h of reaction time in aqueous environment and in absence of side reactions [132]. In a more recent publication SBDC functionalized silicon wafers were used to prepare zwitterionic poly (3-(dimethyl (4-vinylbenzyl) ammonium) propyl sulfonate) brushes with an anti-polyelectrolyte effect via SI-PIMP [133].

Gold surfaces were firstly modified with iniferter groups by Vansco and co-workers who synthesized a disulfide-containing photoiniferter (Figure 10C, DTCA). Surface initiated polymerization from gold is desirable because it is compatible with a broad variety of surface analytical techniques. Furthermore, the Au-S bond can be easily cleaved by treatment in a dilute solution of iodine leading into the release of formerly tethered macromolecules which can then be further characterized [8]. DTCA built a self-assembled monolayer on gold surfaces and was subsequently used to initiate controlled radical photopolymerization from aqueous NIPAM solutions to provide a temperature-responsive surface. Although it is well known that the Au-SH bond is not stable upon UV irradiation (exposure with 254 nm UV light of self-assembled monolayers of alkanethiols on gold and silver leads to photochemical oxidation, generating sulfur trioxide anions and therefore lead to Au-S bond breaking [134]), the group reported successfully controlled photopolymerization using a UV lamp with a 280 nm cut-off filter [135]. The same group later investigated how the composition of the self-assembled monolayer of DTCA on Au influences the morphology and property of the photografted PMMA layer, and also modified PMAA layers with cell-adhesive arginine-glycine-aspartic acid for biochemical applications [136,137]. By growing zwitterionic monomers from a DTCA-functionalized gold surface, Krause et al. developed a coating, which was ultra-low fouling to undiluted human blood plasma, and also showed specific protein detection properties [138].

Another strategy for coupling photoiniferter-groups to different surfaces was shown by Griffete et al., who attached iniferter groups to the surface of iron oxide nanoparticles using aryl diazonium salt chemistry [139]. The coupling mechanism relies on the reduction of the diazonium salt by the metal-surface or reducing agents in solution, generating aryl-radicals that are able to covalently couple to metal or oxide surfaces [140]. This coupling method does not require dry or oxygen free conditions [141]. Diazonium salts provide an excellent alternative to thiols and silanes. While the latter mostly bind to oxidized surfaces, it was proven that Au-aryl bonds have an enhanced stability compared to Au-SH bonds (bond strengths 132.9 and 118.7 kJ/mol, respectively) [75]. Based on this, Griffete et al. designed two different molecules bearing diazonium salt coupling units. One directly carried the coupling agent (a diazonium salt) and the iniferter group (Figure 10B), and the other one contained the coupling agent and a chloromethyl unit (BF_4_, N_2_-C_6_H_4_–CH_2_–Cl), which was reacted with DC-H in a two-step method. Magnetic nanoparticles (iron oxide) with a pH-sensitive PMAA coating were then produced with both coupling reagents [139]. Extending the scope of this technology, the same group successfully functionalized aluminum nanoparticles with photografted PMAA, [140] and improved the colloidal stability of PMAA-coated ultra-small superparamagnetic iron oxide nanoparticles in water. Therefore, oligo(ethylene oxide) spacers of various sizes were introduced between the diazonium salt moiety and the iniferter group of the coupling reagent, which improved the colloidal stability of the nanoparticles in water and ethanol due to the hydrophilic chains [142]. Furthermore, they also modified Au nanorods with a crosslinked MAA and N,N-methylenebisacrylamide copolymer by combining diazonium salt chemistry and SI-PIMP [143].

Table 2 summarizes surface coupled photoiniferters presented in this chapter. The wavy lines in the first column indicate the surface to which the photosensitive group is coupled. The substrate materials as well as the related references are included in columns 2 and 3.

### 3.2. Surface Initiated Atom Transfer Radical Polymerization (SI-ATRP)

**Figure 11:**
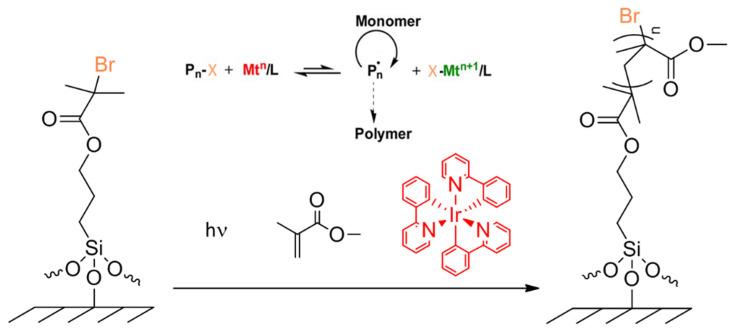
ATRP mechanism and “grafting from” photopolymerization of MMA on a silane-functionalized surface (modified from ref. [144]). The ATRP initiator is labeled in orange, and the photoredox catalyst (Mt^n^/L = Ir(ppy)_3_) is labeled in red.

ATRP is the most frequently used controlled radical polymerization for the synthesis of polymers with adjustable molecular weight, dispersity and architecture. Compared to other controlled radical polymerization techniques, ATRP is chemically versatile and robust [4]. Since its discovery by Matyjaszewski, Sawamoto and Perec in 1995, there has been huge progress in polymer synthesis, metal catalytic systems, scope of polymerizable monomers, industrial applications, etc. The development and mechanisms of ATRP is well described in the literature, especially with respect to the metal catalyst systems (see ref. [144]). This chapter focuses on light triggered ATRP with surface coupled initiators. In traditional ATRP the initiating species is an alkylhalide (labeled in orange), which is activated by a low oxidation state transition-metal (photo)-catalyst (labeled in red), generating an alkyl radical and an oxidized form of the transition-metal complex though halogen atom transfer. The latter one is also referred to as the deactivator. Numerous monomers are added to the alkyl radical or propagating chain before it becomes deactivated by the high oxidation state metal complex [116]. The general mechanism of the ATRP process and a polymerization example with MMA is given in Figure 11. In this figure, Mt^n^/L, labeled in red, represents the photoredox catalyst Ir(ppy)_3_ (tris[2 -phenylpyridinato- C2,N]iridium (III).

**Scheme 15:**
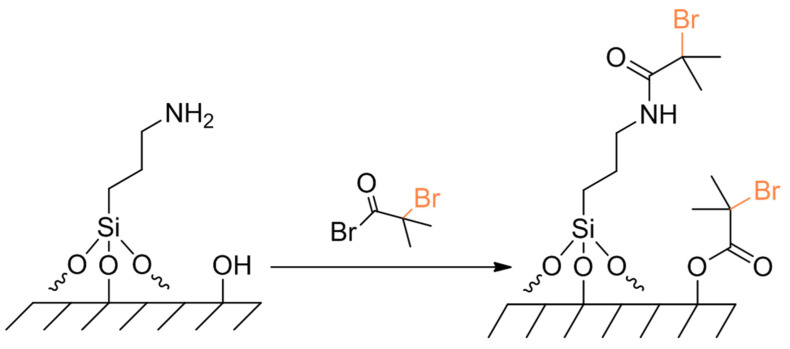
Coupling of an ATRP initiator (orange) to an amino-functionalized surface.

To give one recent example of an application, Zholdassov et al. used a photochemical printer, equipped with a digital micromirror, capable of rapidly elucidating the kinetics of light induced SI-ATRP of different monomers in order to develop patterned “5D and 6D hypersurfaces” with tailored polymer brush-heights, chemical compositions and changes upon an external stimulus. They impressively demonstrated how to encrypt data within those hypersurfaces using stimuli-responsive polymer brushes surrounded by nonresponsive brushes. In doing so, they firstly introduced amino groups to silicon substrates by silanization with APTES, followed by the treatment with α-bromoisobutyryl bromide, leading to alkyl halide species that served as the ATRP initiator (see Scheme 15). Photopolymerization of NIPAM and DMAAm was carried out in the presence of tris[2-phenylpyridinato- C2,N]iridium (III) (Ir(ppy)_3_) as the photocatalyst [145].

Many other examples of SI-ATRP derivatized polymer brushes on different surfaces are given in the reviews of Chen and co-workers [21] and Barbey et al. [4]. However, novel metal-free catalytic systems are sparsely, or even not, described in those contributions and will be outlined herein. The use of organic photoredox catalysts (PC) such as 10-phenylphenoxanine or 10-phenylphenothiazine extended the use of light-induced SI-ATRP for biomedical and electronic applications [146]. A proposed mechanism involves the photoexcitation of PC to an excited state, which is employed to reduce alkyl bromides via an oxidative quenching pathway, generating a carbon centered radical for the polymerization and the radical-ion pair PC*^+^Br^−^. The latter one deactivates the propagating chain, leading to recovered alky bromide and PC, which can be re-initiated upon UV irradiation [147].

Ma et al. used the same initiator-coupling strategy (silanization with APTES and subsequent treatment with α-bromoisobutyryl bromide, Scheme 15) to modify mesoporous silica materials with polymer layers derived from SI-ATRP of MMA, NIPAM and DMAEMA using 10-phenylphenothiazine as a photoredox catalyst. The obtained hybrid material (PMMA modification) showed enhanced adsorption ability for toluene as a representative example [148].

Another photoassisted SI-ATRP approach was demonstrated by Zeng et al. [149], who immobilized an ATRP initiator onto the surface of nanodiamonds via direct esterification between superficial hydroxyl groups and α-bromoisobutyryl bromide (Scheme 15). Surface initiated photopolymerization with 10-phenylphenothiazine of 2-methacryloyloxyethyl phosphorylcholine resulted in a biocompatible surface and enhanced the nanodiamonds’ dispersibility in various solvents. Conventional esterification reactions employing carboxylic halides and anhydrides [150] have also been used for the surface functionalization of wood [151] and cellulosic materials [152].

Zeng and colleagues also developed an ATRP initiator for the surface functionalization of Eu^3+^ doped luminescent hydroxyapatite (HAp) nanorods. First, NH_2_-containing HAp was prepared via ligand exchange reaction using adenosine monophosphate as ligand. The amino groups were subsequently reacted with α-bromoisobutyryl bromide yielding a photoreactive surface from which (ethyleneglycol) methylether methacrylate was grafted, generating hydrophilic luminescent HAp nanorods [153].

**Figure 12:**
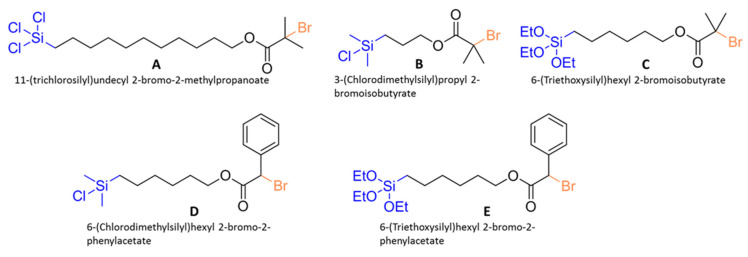
Different compounds containing both an ATRP initiator (orange) and a coupling moiety (blue).

Diszeciki et al. used the same inexpensive organic phenothiazine-based photocatalyst to grow patterned (co)-polymer brushes of MMA, 2,2,2-trifluoroethyl methacrylate and 2-(methylthio)ethyl methacrylate from Si-wafer surfaces and silica nanoparticles using visible light (405 nm). Therefore, they synthesized a compound which comprised a trichloro-silane and an initiator moiety (Figure 12A), which was coupled to the corresponding surface via silanization [154]. In a continuative and comparative study, Yan. Et al. pointed out that surface-tethered ATRP initiators derived from ethyl 2-bromo-2-phenylacetate are much more reactive than previously used ethyl 2-bromoisobutyrate-derived structures, thus leading to a higher grafting density. In this context they synthesized four ATRP-initiators by combining the two mentioned structures with chloro- and alkoxy silane anchors (Figure 12B–E) and coupled them to silica nanoparticles of different sizes. Surface photopolymerization of MMA was carried out using 10-phenylphenothiazine as photocatalyst [146].

To conclude this sub-chapter, clickable initiators can also be used for the combination with different types of controlled radical polymerizations for preparing highly functional tailor-made macromolecules. Just to name a few, alkyne and ene-functionalized initiators, azide-functionalized initiators and maleimide-functionalized initiators for pre or post modifications are included. More detailed information can be found in reference [155].

### 3.3. Surface Initiated Photoinduced Electron/Energy Transfer Reversible Addition-Fragmentation Chain Transfer Polymerization (SI-PET-RAFT)

**Figure 13:**
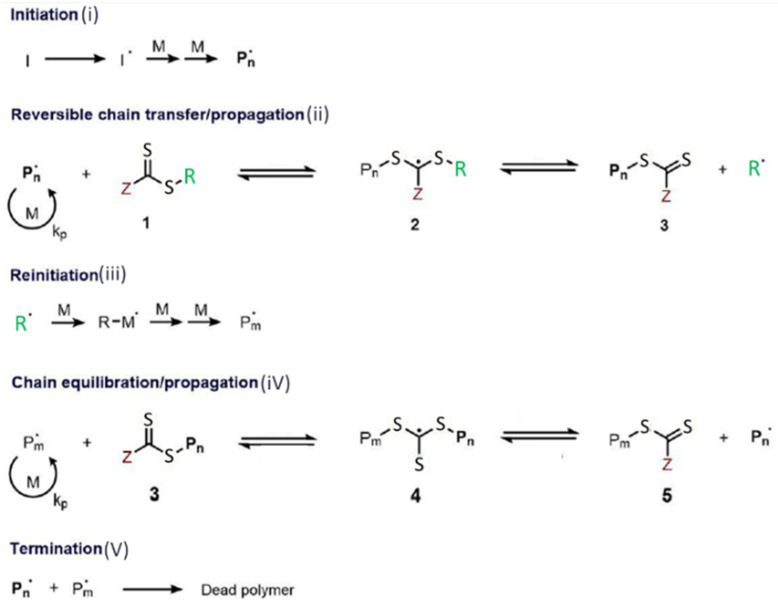
General mechanism of RAFT polymerization (slightly modified from ref. [156]).

The RAFT polymerization technique was introduced in 1998 by Chiefary et al., and is a versatile method for the controlled synthesis of functional polymers in different reaction conditions (aqueous or organic solutions, bulk, emulsion and dispersion) [156]. The living character relies on a simple and accessible class of organic materials containing a thiocarbonylthio moiety, which is capable of reversibly reacting with propagating chains via a chain transfer mechanism leading into the dormant (end-capped) species. Switching between the propagating and the dormant state suppresses terminating reactions and therefore provides control over the molecular weight and dispersity of the polymer. The general mechanism is shown in Figure 13. The first step includes an initiating step (i), typically done by an initiator, followed by chain propagation. The propagating radical (•P_n_) reacts (ii) with the so-called RAFT agent (**1**) and forms radical **2**, which then decomposes to compound **3** and the radical R• (for subsequent re-initiation (iii)). Compound **3** can react with another propagating radical (•P_m_), giving radical **4**, which then releases the radical •P_n_ (iv). The most important aspect of the RAFT process is the rapid equilibrium (iv) between the two propagating radicals, •P_n_ and •P_m_, and dormant species, **5** and **3**, via intermediate **4**. This provides equal opportunities for all chains to grow, thus leading to low polydispersity. The rapid interchange within this equilibrium also limits termination reactions by recombination of •P_n_ and •P_m_ (v). The chemical structure of the substituents R and Z (colored green and red in Figure 13) and the monomer choice is crucial for successful RAFT processes [156,157]. More detailed information on the chemical structure of RAFT agents and the resulting properties can be found, e.g., in ref. [158].

Recently, light induced RAFT processes have attracted much attention because of the well-known advantages of light, including the spatial and temporal control of the polymerization and the possibility to act at low temperatures [159]. In comparison to conventional RAFT polymerizations, in which the processes are initiated by a thermal initiator such as AIBN or benzoyl peroxide, light-triggered RAFT polymerizations require a photoredox catalyst (PC) and provide several advantages. First, a very low amount of PC is needed (ppm range) and the polymerization can be carried out in the presence of molecular oxygen, which provides the possibility to translate polymerization processes from controlled environments such as gloveboxes to the benchtop. Commonly used PCs which have been reported in the literature are transition-metal complex photocatalysts (for example, fac-Ir(ppy)_3_, Ru(bpy)_3_Cl_2_, ZnTPP), eosin Y, porphyrines and metal oxides [12,156,159]. Since the pioneering work referred to PET-RAFT in 2014, the interaction between an excited state PC and the RAFT agent remains uncertain and challenging. A comprehensive article by Allegrezza et al. focuses on this topic. In general, possible mechanisms involve electron or charge transfer processes from the excited PC to the RAFT agent, both yielding a radical (•R or •P_n_) which initiates the RAFT process [160].

In this sub-chapter some examples of surface tethered RAFT agents for subsequent surface initiated photopolymerizations are given. However, it can be concluded from above that a surface coupled RAFT agent does not constitute a surface coupled photoinitiator. Therefore, only some recent examples are shown in this manuscript.

**Figure 14:**
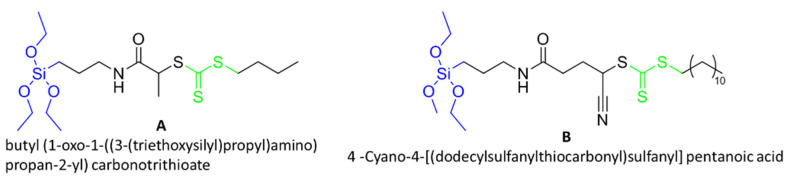
RAFT agents (green) with organosilane anchor moieties (blue).

Ng et al. used oxygen tolerant SI-PET-RAFT for the synthesis of homo-, block- and gradient polymer brushes on glass slides, employing a broad range of monomers (acrylamides, methacrylates, acrylates) to develop antifouling surfaces. The RAFT agent was immobilized using silanization chemistry. Thus, they synthesized two different RAFT agents, which carried a silane anchor moiety (see Figure 14A,B). Visible light mediated photopolymerization was carried out using a zinc-porphyrin PC (5,10,15,20-tetraphenyl-21H,23H-porphine zinc). Because of the lower experimental effort, which results from the oxygen tolerance of SI-PET-RAFT (absence of inert gas, vacuum, etc.), the authors pointed out that this benchtop methodology provides a high throughput approach for the identification and evaluation of polymer brush structure-property-performance relationships [12]. One year later the same group prepared a polymer brush with antifouling and visible light triggered bactericidal properties, on circular glass substrate using the same bifunctional RAFT agent (Figure 14A) [161].

**Scheme 16:**
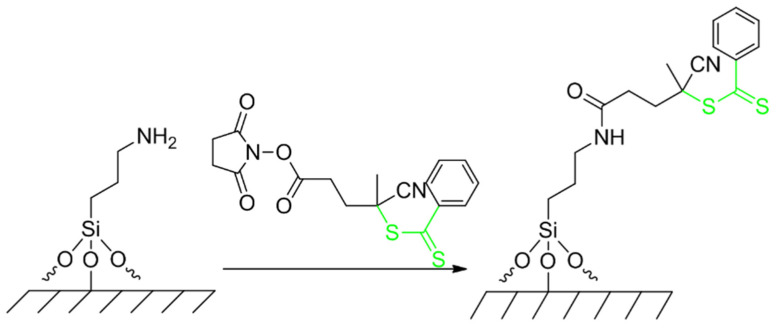
Coupling strategy for the introduction of the RAFT agent (green) to surfaces.

Staying with anti-fouling surfaces, Kuzmyn et al. used SI-PET-RAFT and eosin Y and triethanolamine as catalysts to polymerize three different monomers (oligo(ethylene glycol) methacrylate, N-(2- hydroxypropyl)methacrylamide and carboxybetaine methacrylamide) from silicon surfaces that were functionalized with a RAFT agent. A mechanism of that photopolymerization, triggered with visible light, was proposed by Xu et al. in 2015 (ref. [162]). The reaction may proceed via a reductive quenching cycle of eosin Y in which the amine acts as a sacrificial electron donor to reduce oxygen, allowing for the polymerization system to proceed under atmospheric conditions. To immobilize the RAFT agent, silicon substrates were firstly modified with APTES, yielding surface coupled amino groups. In the next step, the amino groups were exposed to 4-cyano-4-(phenylcarbonothioylthio)pentanoic acid N-succinimidyl ester in the presence of triethylamin as a catalyst, leading to surface tethered RAFT agents (see Scheme 16) [163]. A similar coupling strategy was then used by the same group in a later publication, modifying gold surfaces with cysteamine, followed by the reaction, which was previously mentioned (see Scheme 16). The group pointed out that SI-PET-RAFT with eosin Y and triethanolamine as catalyst is a scalable, robust, mild, oxygen-tolerant and heavy-metal-free method for the synthesis of antifouling (co)-polymer brushes on gold surfaces [164].

A different coupling strategy to immobilize a RAFT agent onto the surface of cadmium selenide (CdSe) quantum dots was recently presented by Egap et al. CdSe is a novel class of PC because of the unique electrical and optical properties based on quantum confinement effects. Two RAFT agents were immobilized via ligand exchange between the oleic acid capped quantum dot surface and the bithiol group of the RAFT agent. Organic-inorganic polymer-coated quantum dot nanocomposites were obtained in a “grafting from” approach, where the quantum dots act as the PC and as the building block at the same time. These reactions were carried out with different methacrylates under blue light exposure (460–480 nm) in the presence of diisopropylethylamine as an electron donor. The advantages of this method are (i) oxygen tolerance, (ii) low catalyst loading, (iii) one pot synthesis and (iv) the simplicity in tuning the quantum dot properties [165]. A similar process for RAFT agent immobilization, through ligand exchange, with subsequent growth of polymer brushes onto lanthanide-doped up-conversion nanoparticles was conducted by Hu et al. PMMA layers were obtained after near infrared mediated RAFT polymerization. The authors pointed out that this efficient one-pot “grafting from” process could be also conducted in a vessel, which is shielded by a biotissue barrier due to the good penetration performance of the near infrared laser [166].

**Scheme 17:**
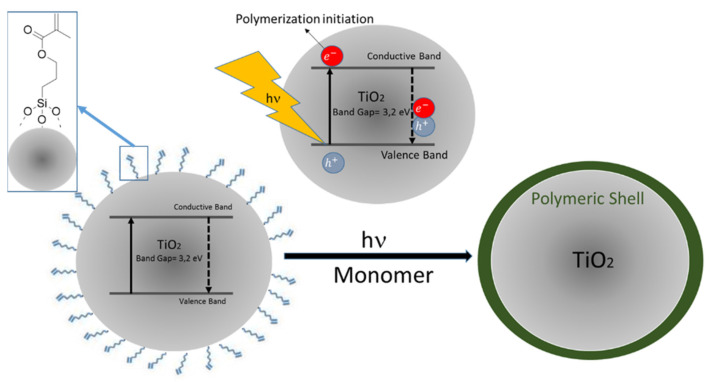
Generation of inorganic/polymer nanocomposites via self-catalytic photopolymerization of pre-modified TiO_2_ particles [169,170].

Finally, one example of photocatalytic surface-initiated polymerization is presented, which does not rely on SI-ATRP, SI-PIMP or SI-PET-RAFT mechanisms, but relies on self-initiation. Titanium dioxide (TiO_2_) is a photocatalyst, which has been widely applied for photovoltaics, nanoscience, biomedical fields and for the oxidative degradation of harmful organics upon light irradiation [167,168]. Excitation of TiO_2_ with light in the range between 388 and 414 nm transfers electrons (labeled in red in Scheme 17) from the valence band to the conductive band, generating electron hole pairs as illustrated in Scheme 17. The free electrons as well the positive charge carriers migrate towards the surface and react with chemicals in the vicinity of the surface [169]. This was exploited by Wang et al. to produce core/shell composite nanospheres. To ensure covalent linkage between the TiO_2_ core and the polymeric shell, the particle surface was pretreated with 3-(trimethoxysilyl)propyl methacrylate, yielding surface coupled methacrylic functionalities (Ti-O-Si bond). These C=C bonds are expected to capture electrons generated by TiO_2_ upon UV excitation, thus starting the polymerization reaction. With this method a PS and PMMA shell was grown from the TiO_2_ particle surface as shown in Scheme 17 [170].

## 4. Coupled Photoacids and Photoacid Generators

Radical photopolymerization, which has been covered in the previous chapters, exhibits some drawbacks. Firstly, oxygen inhibition by atmospheric oxygen can inactivate the propagation reaction. Secondly, radical photopolymerization suffers from volume shrinkage due to irradiation, which can have a severe influence on the structural material properties and, last but not least, the choice of monomers is generous but limited. Polymerizations, based on nucleophilic substitutions, including most ring opening mechanisms, as well as step growth polymerizations, cannot be initiated by radicals. Therefore, the photogeneration of a base or acid as catalyst is desirable [171].

**Scheme 18:**
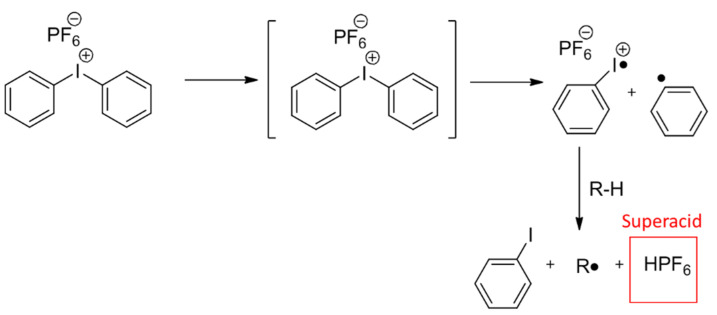
Photolysis mechanism of a photoacid generator as described by Crivello [171].

The phenomenon of photoacidity has been extensively studied since the pioneering work of Förster and Weller in the middle of the last century. Generally, there are two types of photoacids: photoacid generators (PAGs) and photoacids (PAHs). Photoacid generators, such as the prominent Crivello salts (first introduced in 1970) typically undergo irreversible cleavage reactions generating a strong Brönsted acid under UV irradiation (<300 nm). The mechanism is shown in Scheme 18 [171].

In contrast to PAGs, typical photoacids (PAHs) including aromatic alcohols, such as phenols, naphthols and pyrenols, do not decompose upon UV irradiation, but increase their acidity in the excited state. Upon UV irradiation of aromatic alcohols, an electron is removed from the non-bonding orbital located at the oxygen atom, and transferred into the lowest unoccupied molecular orbital, which is generally not located at the oxygen atom. Therefore, the electron density and—as a consequence—the basicity of the oxygen atom is reduced significantly. To give an example, the acidity of 2-naphthol becomes 10^7^ times higher as a result of UV excitation [172]. In a similar way, a photobase undergoes an increase of the pKa value upon excitation, and excited state proton capture upon UV radiation is observed. A fundamental difference between PAG and PAH is that in the case of PAG the acidic moiety (i.e., a Brönsted acid such as HPF_6_) remains after termination of UV irradiation, which gives rise to so-called dark reactions. In contrast to this, the increased acidity of a PAH (e.g., pyrenol) vanishes when the light is cut off, because it recombines with the proton reaching its former state [173,174].

This chapter is dedicated to surface coupled photoacids and photobases as well as photoacid generators. Surface tethered photobase generators have not been described in the literature as yet.

**Scheme 19:**
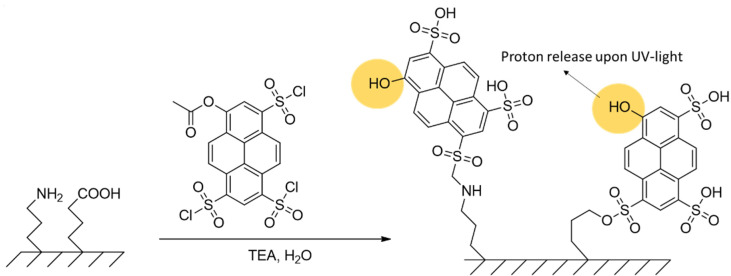
Coupling strategy for a photoacid onto a surface.

**Scheme 20:**
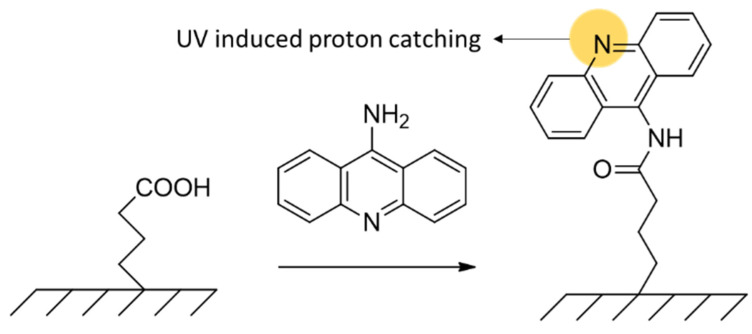
Coupling strategy for a photobase onto a surface.

Quite recently (2021) and for the first time, Amdursky et al. explored the capability of biopolymer-tethered photoacids and photobases to conduct excited state proton transfer and capture, respectively. The authors used pyranine (8-hydroxypyrene-1,3,6-trisulfonic acid) as a Brönsted photoacid with a pK_a_-value of approximately 7.4 in the ground state and 0.3–1.3 in the excited state. To attach this molecule to the surface of electrospun mats, made of bovine serum albumin (BSA) proteins, the molecule was firstly reacted with thionyl chloride generating a triple electrophilic sulfonyl chloride derivative, which was attacked by amines and hydroxy groups from the surface, leading to sulfonamides and sulfonate esters, respectively (see Scheme 19). In a second part of the study, 9-aminoacridine as a photobase (pK_a_ of 10.7 in the excited state) was employed to be coupled to the same surface. This molecule was coupled utilizing the reaction between amino groups of the photobase and carboxylic groups of the surface in the presence of a carbodiimide as a coupling agent, forming amide bonds (see Scheme 20). The results showed a change in the measured ionic conductivity upon UV irradiation, due to excited state proton transfer/capture of the surface tethered photoactive species [175].

Surface immobilized PAGs were described by Dai et al. in a recent US patent [173]. This strategy is based on PAGs which contain a polymerizable unit in the anion. Characteristic examples are (triphenylsulfonium) 3-sulfopropyl methacrylate and (triphenylsulfonium) 2,3,5,6-tetrafluoro-4-(methacryloyloxy)benzene, with both anions being derivatives of methacrylic acid. Those monomers were copolymerized with methacrylates bearing anchor units, such as glycidyl methacrylate or 3-(trimethoxysilyl)propyl methacrylate. The resulting copolymers, which comprised the PAG and the anchor unit were introduced to the surface of a semiconductor. The immobilized PAGs can then be used to pattern materials coated on top of the immobilized PAGs, allowing for direct patterning without the use of a photoresist, thereby reducing process steps and cost.

## 5. Photocoupling and Photoclick Reactions

Since the beginning of modern chemistry, reactions which are accompanied by few or no side-reactions, high yield and well-defined linking reactions, are desired. For this, Sharpless et al. established the concept of so called “click reactions” in 2001. These reactions include: (i) cycloadditions (1,3- dipolar Huisgen-cycloadditions, Diels–Alder reactions), (ii) nucleophilic ring-opening of stressed electrophiles such as epoxides, (iii) reactions of non-aldol carbonyl compounds and (iv) the addition to carbon-carbon multiple bonds (in particular thiol-ene chemistry and Michael additions). Light induced click reactions combine the advantages of above mentioned click reactions with the benefit of photoinduced “grafting to” processes. The most important benefits are the temporal and spatial control of light-induced stimulation [5]. In this chapter, some prominent light induced click reactions between (polymer-) surface coupled initiating groups and different (polymer-) compounds in terms of the “grafting to” approach will be reviewed. It has to be mentioned that some reactions presented in the following do not always meet the strict conditions of click reactions. However, this does not mitigate their importance.

### 5.1. Coupled Azide

Copper(I)-catalyzed azide-alkyne cycloaddition (CuAAC) between azides and terminal alkynes (developed by the Sharpless and Meldal groups) is an efficient, versatile and regiospecific click reaction which proceeds under mild conditions. There is more than one possibility to start the reaction, including direct and indirect photolysis. With respect to direct photolysis, a ligand coordinating Cu(II) center absorbs UV light, resulting in intramolecular electron transfer and the conversion of Cu^II^ into Cu^I^ [5]. However, contributions found in the literature mainly include the indirect approach to catalyze azide-alkene click reactions including a free photoinitiator. Since the photoinitiator was not coupled to surfaces in those studies, these examples will not be presented in detail here [176,177,178].

**Scheme 21:**
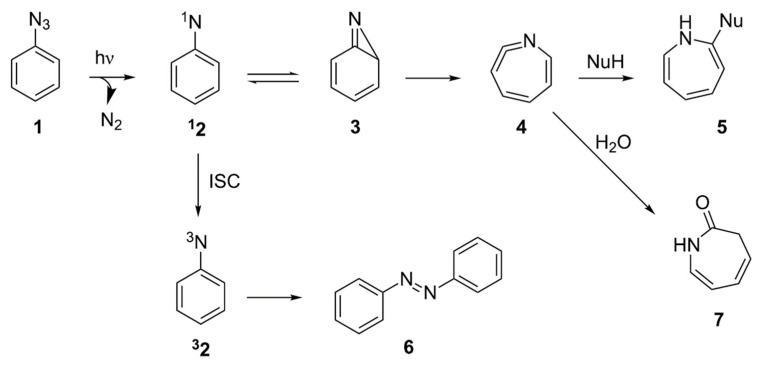
Phenyl azide photochemistry (taken from ref. [180]).

Phenyl azides are popular photosensitive compounds because of their high efficiency, fast reaction kinetics, high storage stability and their simplicity in synthesis [5]. Upon UV irradiation, phenyl azides (**1**) are converted into singlet nitrene (^1^**2**), a highly reactive intermediate, by splitting off molecular nitrogen. This singlet nitrene can either reach its triplet state (^3^**2**) via intersystem crossing (followed by further reaction pathways such as dimerization to diazo compounds (**6**)), undergo insertion or addition reactions with nucleophiles and rearrange (ring expansion) to form benzazirine (**3**), which in turn rearranges to didehydroazepine (**4**). The didehydroazepine itself readily reacts with secondary amines and other nucleophiles yielding the corresponding azepine (**5**). If the reaction is performed in water, 3H-azepinone (**7**) is typically generated. These reactions are summarized in Scheme 21. Generally, the product distribution of particular aryl azides is dependent on the experimental conditions and the substituents which are attached to the aromatic ring (electron-withdrawing groups in para position to the nitrene lead to insertion and less rearrangement reactions) [179,180].

**Figure 15:**
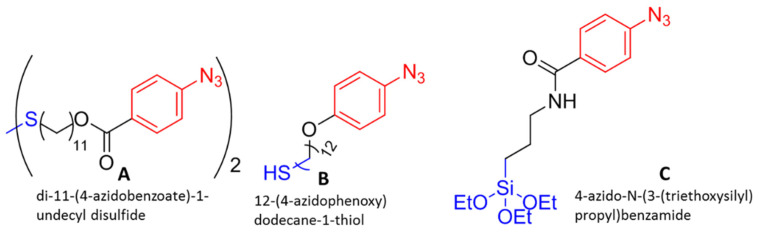
Phenyl azides (red) with anchor moieties (blue).

Photosensitive self-assembled monolayers of phenyl azides were covalently coupled to gold surfaces by Wollmann et al. in 1994, who synthesized a compound bearing a phenyl azide and a disulfide functionality (Figure 15A). The reaction of excited phenyl azides with different secondary amines was exploited to form Au-confined derivatives of 3-H azepine and hydrazine. Furthermore, they demonstrated photopatterning by using azido functionalized Au surfaces and a liquid amine between the mask and the substrate [181]. About twenty years later, phenyl azide modified gold nanoparticles were produced by Snell et al., who synthesized a compound bearing both a phenyl azide and a thiol moiety (Figure 15B). The high reactivity of the nitrenes, generated from the azide, was employed to tether the AuNP to the native surface of carbon nanotubes, reduced graphene oxide and micro-diamond powder to yield hybrid materials [182].

**Scheme 22:**
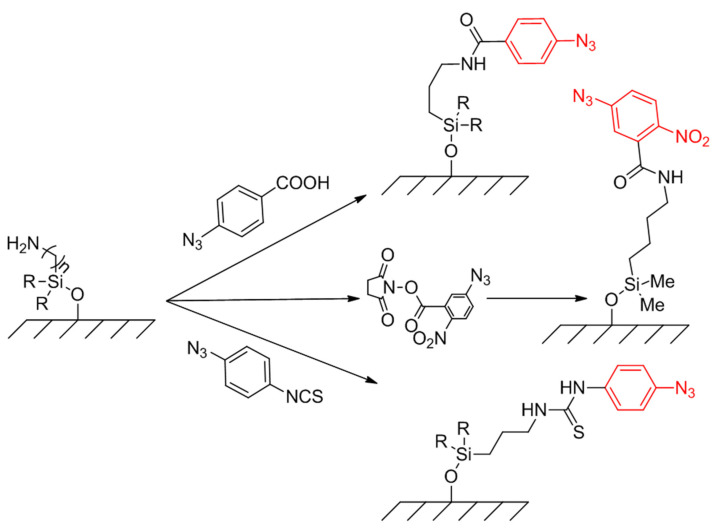
Coupling strategies for phenyl azide (red) immobilization.

The preparation of ultrathin and hydrophilic polymeric cushions on glass slides and Si-wafer surfaces was done by Elender et al. Firstly, the corresponding surface was modified with amino-groups by silanization with 4-aminobutyl-dimethyl-mono-methoxysilane, followed by the coupling of the phenyl azide moiety on the surface using N-5-azido-2-nitrobenzoyloxysuccinimide (see Scheme 22). Upon UV radiation, azide groups were converted into nitrenes which performed insertion reactions with hydroxyl groups from dextran achieving the covalent linkage of a reversibly swelling dextran film, which was found useful for biosensorics [183].

Azido terminated silica nanoparticles were prepared by Zhao and co-workers through upstream surface-modification of virgin silica nanoparticles with amino groups via silanization with APTES, followed by reaction with 4-azidobenzoic acid forming an amide bond (Scheme 22). To overcome the problem of small contact between spherical nanoparticles and the solid substrate during the immobilization, poly (allylamine hydrochloride) was also functionalized with phenyl azido groups via the reaction between side-chain amino groups and 4-azidobenzoic acid in the presence of a carbodiimide as coupling agent. After a layer-by-layer assembly, a tailored and crosslinked network was established among the silica nanoparticles, the surrounding polymer chains, and the substrate (O_2_-plasma treated cotton fabrics) under UV irradiation, utilizing azide photochemistry. The authors pointed out that this strategy may be suitable to form stable nanoparticle coatings on almost any organic substrate [184]. Staying with silica nanoparticles, Kern and colleagues prepared phenyl azide modified SiO_2_ nanoparticles via the reaction between surface tethered amino groups and 4-azidophenyl isothiocyanate (Scheme 22). Photo-crosslinking experiments with poly (norbornene dicarboxylic acid, dimethyl ester) and polyisoprene revealed that these particles act as non-migrating photoinitiators. Moreover, the successful immobilization of the nanoparticles on virgin PET, polyethylene and polyamide surfaces upon UV radiation has been demonstrated [185].

Recently, Picu et al. developed epoxy nanocomposites, which are reinforced with silica nanoparticles, also bearing phenyl azide groups at the surface [186]. In this context the mechanical properties of the filler-matrix-interface was controlled upon light exposure. For this, an azidophenyl-silane was synthesized (Figure 15C) and grafted onto the surface through silanization. The received particles were mixed into epoxy resin systems followed by thermal curing. Cured samples containing the photosensitive filler displayed a strong decrease in toughness upon UV illumination. This photo-embrittlement results from the covalent coupling between the silica nanoparticles and the epoxy resin matrix, generating a strong interface. This is one of the few examples where the (photo)triggered change from a weak to a strong interface in a composite was evidenced by mechanical analysis.

**Figure 16:**
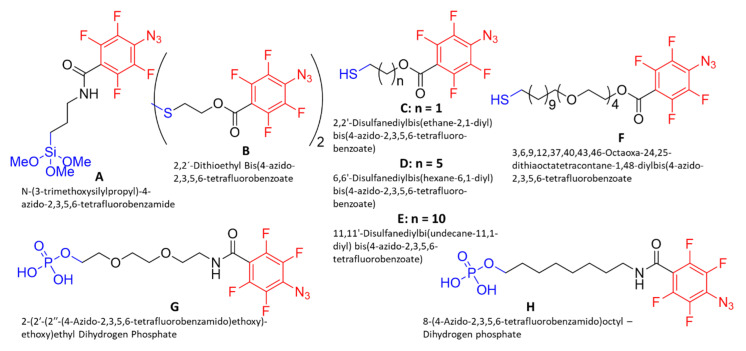
Perfluorophenyl azides (red) with anchor moieties (blue).

Significant work was done by Yan and co-workers, who performed intense research on perfluorophenyl azides (PFPA) which were applied as photosensitive coupling agents in surface functionalization and nanomaterial synthesis. An important finding in the photochemistry of PFPA was that halogen atoms such as F or Cl on the aromatic ring significantly increased the lifetime, and suppressed ring expansion reactions of the singlet nitrene species. Therefore, the extent of insertion reactions was increased which was decisive to graft organic materials. Nitrenes generated from PFPA reacted with CH, NH and C=C bonds, so that the coupling chemistry is suitable for a broad range of molecules regardless of their structures, architecture and properties [7]. In some proof-of-concept experiments, SiO_2_ surfaces were modified with a synthesized PFPA-trimethoxysilane (Figure 16A). The UV grafting of PS and PEOX to the surface was carried out after spin casting and followed by solvent extraction [187,188,189,190]. The UV-induced immobilization of highly crystalline materials such as isotactic PP and low molecular weight PEG did not work well with this method, since efficient insertion reactions require a close contact between the solid polymer and the surface azido groups. In this case, thermal activation was applied, melting the polymer film to increase surface-polymer interaction and simultaneously trigger the cleavage of the azido group [7,191].

Aryl azide chemistry has also been used to generate biocompatible surfaces based on carbohydrates. Carbohydrates received high attention because of their interactions with proteins and other biological entities. Therefore, Pei et al. synthesized a novel compound, carrying a PFPA as well as a disulfide moiety (Figure 16B), which was able to build a self-assembled monolayer on Au. This PFPA-derivatized surface was subsequently used to tether a thin layer of PEG upon thermal activation, because of the above-mentioned reasons. Carbohydrate-derivatized surfaces for selective protein binding were achieved, using different PFPA-derivatized carbohydrates, which coupled onto the surface upon UV irradiation via insertion reactions [192]. The scope of PFPA derivatized surfaces in biomedical applications was extended by the synthesis of four thiols (Figure 16C–F) with different chain lengths (spacer) containing hydrocarbon and ethylene oxide units between the thiol and the PFPA functionality. Gold nanoparticles were modified with these compounds followed by UV-coupling of different carbohydrates to study their interaction with proteins (binding affinity) depending on the size of the nanoparticle, spacer length, PFPA-density, etc. [193] The photochemical properties of PFPA were also employed to produce thin PEOX films on amino-functionalized glass slides by Pei et al. A monolayer of PFPA moieties was achieved by treating the aminosilylated glass slides with N-hydroxylsuccinimide-derivatized PFPA which was then irradiated with UV light after the PEOX film had been deposited. In the end, spatially addressable carbohydrate microarrays were produced through insertion reactions of PFPA derivatives in carbohydrates upon UV irradiation [194]. To modify (magnetic) iron oxide nanoparticles with carbohydrates for sensor applications, Yan and colleagues also synthesized two phosphate-functionalized PFPAs (Figure 16G,H), which can both react with superficial hydroxyl groups to form a stable Fe-O-P bond [195].

An efficient photocoupling agent, based on PFPA conjugated polyallylamine, was developed by Kuba et al. for the photo-induced immobilization of polymers (PS, PEOX, poly(vinyl pyrrolidone)), nanoparticles (PS fluorescent silica nanoparticles), graphene and small molecules (carbohydrates) on Si-wafers, which were pre-modified with epoxy groups at the surface via silanization with 3-glycidyloxypropyltrimethoxysilane. The photocoupling agent was received through the reaction between amino groups of polyallylamine hydrochloride and N-hydroxylsuccinimide-derivatized PFPA. The remaining amino groups were then used to covalently couple the reagent to the Si-wafer surface via the reaction with epoxides. The authors claimed a higher immobilization efficiency compared to PFPA-silane functionalized surfaces [196].

### 5.2. Coupled Tetrazole

**Scheme 23:**
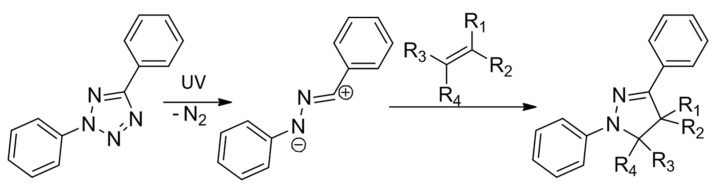
Schematic illustration of the photo-induced formation of the nitrile imine from tetrazole and following 1,3-dipolar cycloaddition with dipolarophiles [198].

The concept of light-induced 1,3-dipolar cycloaddition of a tetrazole and an alkene was firstly shown in 1967 by Huisgen and Sustmann. Upon UV irradiation (302 nm) the tetrazole moiety decomposes by splitting off molecular nitrogen, leading into a highly reactive nitrilimine-intermediate. This intermediate, as can be seen in Scheme 23, readily reacts with electron-poor terminal alkenes such as (meth)acrylics, fumarates and alkynes through a 1,3-dipolar-cycloaddition forming stable, fluorescent pyrazoline products. The advantages of this chemistry include high quantum yield and reaction rate, the benefit that no metal catalysts are required and that this reaction is biorthogonal [5,197].

**Figure 17:**
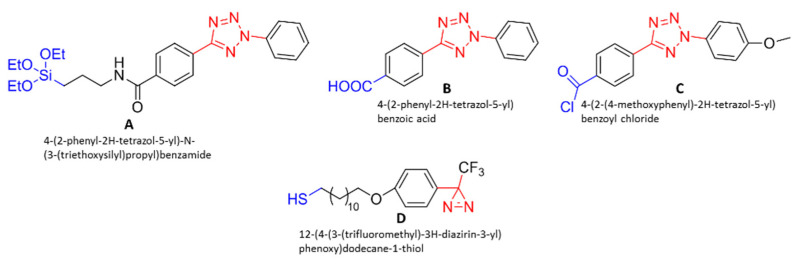
Tetrazole and diazirine compounds (red) which can be covalently coupled to surfaces using anchor moieties (blue).

The nitrile imine-mediated tetrazole-ene cycloaddition reaction (NITEC) was exploited to graft polymers onto inorganic (silicon) and bioorganic (cellulose) surfaces, forming a highly fluorescent linkage under ambient conditions by Dietrich et al. Covalently coupled tetrazole moieties at the surface of cleaned and activated silicon wafers were obtained via silanization with a tetrazole silane (Figure 17A). Subsequently maleimide-functionalized polymers were utilized to perform the NITEC reaction, leading to a surface tethered polymer film. Furthermore, within the same contribution, the authors synthesized a carboxy-containing tetrazole (Figure 17B), which was coupled to the surface of cellulose materials via esterification. After grafting of the same maleimide containing polymers (maleimide served as the dipolarophile), highly fluorescent and patterned cellulose sheets were obtained [198]. More recently, interfaces with precise control of cell adhesion were produced utilizing the NITEC reaction. Thus, a novel diaryltetrazole-initiator with an acid-chloride moiety (Figure 17C) was synthesized, including an electron donating methoxy group in para-position to the N_2_-phenyl ring increasing the reactivity of the nitrile-imine dipole within the NITEC mechanism. Furthermore, the methoxy group shifted the absorption spectrum from the UVC to UVA region (approx. 320 nm) allowing for the functionalization of more sensitive biosystems. The photoinitiator was coupled to amino groups at polydopamine surfaces, followed by photo triggered NITEC of a maleimide-functional ATRP-initiator. Surface initiated photopolymerization of oligoethylene glycol methyl ether methacrylate leads to patterned cell-repellent surfaces [199].

### 5.3. Coupled Diazirine

Aryldiazirine compounds are well-known photocoupling agents, which were first described as photoaffinity probes in 1973 by Smith and Knowles. Among them, 3-aryl-3-(trifluoromethyl)diazirine received attention because of its high thermal and chemical stability and, in addition, its advantageous photochemical properties. Upon exposure to UV light the diazirine species generates a highly reactive carbene intermediate (by splitting of molecular nitrogen) which can undergo insertion reactions with X-H groups (X=C,O,N) or addition reactions with alkenes. Furthermore, an isomerization leading to azo-compounds is also observed [200,201].

Ismaili and co-workers extensively used diazirine photochemistry to couple diazirine modified Au nanoparticles onto hydroxylated diamond powder via insertion of hydroxyl groups. In a similar way, carbon nanotubes were functionalized via an addition reaction to the π-conjugated carbon skeleton. Furthermore, reduced graphene and glass were functionalized via addition and insertion reactions. As an example, Au nanoparticles were modified using a thiol bearing diazirine derivative (Figure 17D) [201,202,203,204].

Kanoh et al. employed diazirine modified glass slides to immobilize structurally distinct and small molecules with specific protein-binding abilities by UV irradiation. For this concept, the authors prepared a linker bearing both an amino group (as anchor) and the photosensitive diazirine moiety. For preparing photosensitive surfaces, amino functionalized glass slides were treated with N,N-disuccinimidyl carbonate to yield succinimidyl groups at the surface. These groups were then reacted with the diazirine compound, thus providing diazirine units covalently coupled to glass [205].

## 6. Summary and Outlook

In this review it has been shown that the attachment of photoinitiators and other photolabile units to surfaces is a versatile tool, in most cases aiming at the functionalization of surfaces. The large variety of initiators available nowadays provides different possibilities, taking into account the wavelength of excitation, the avoidance of unwanted side reactions and the strength of the generated bonds. Generally, the employed substrate governs the coupling reaction for immobilization of the initiator. Consequently, silane chemistry is found most often with respect to oxidic surfaces of metals and ceramics, and for surfaces of gold, silver and copper thiol chemistry has been utilized exhaustively.

In most cases, light-initiated surface reactions are employed to cover surfaces with polymeric layers by “grafting-from” approaches, to attach functional surface layers onto (nano)particles, to confer hydrophilicity and antifouling properties onto plastic surface, and to achieve the covalent coupling of different phases. Nanoparticle immobilized initiators enable the initiation of photopolymerization by non-migrating photoinitiators, and it has been shown that they can outperform their parent compounds.

The utilization of UV and visible light is of general advantage when mild reaction conditions are required, when thermally sensitive materials such as biomaterials have to be processed and when the reaction has to be performed in a very short interval.

Whilst most of the surface coupled initiators belong to the group of free radical initiators, comparably little research has been performed with surface immobilized photoacid and photobase generators. This is remarkable as it would provide the possibility to generate acidic (or basic) surfaces under UV irradiation, and to initiate cationic or anionic polymerizations at surfaces. This is a certainly a field which has to be unlocked in the future.

## Figures and Tables

**Table 1:** Summary of surface coupled photoinitiators and co-initiators.

Photoinitiator	Substrate	Reference
Type I photoinitiators		
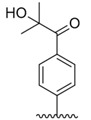	Silicon substrate	[27,28,29]
	Silica nanoparticles	[31,32]
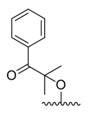	Silica nanoparticles	[33]
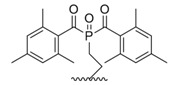	Cellulosic material	[34]
	Cellulose nanocrystals	[41]
	Glass surface	[36]
	Silica microparticles	[37]
	Silica nanoparticles	[32,35,38]
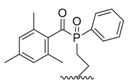	Silica nanoparticles	[31]
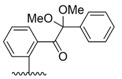	Silica nanoparticles	[39]
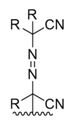	Titanium oxide, ferrite	[42,43]
	Ferrite	[42]
	Silica particles	[42,43,44,46,47]
	Silicon substrate	[46,48,49]
	Gold	[48,50,51,52,53,54]
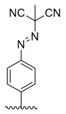	Silicon substrate	[48]
	Gold	[55]
Type II photoinitiators		
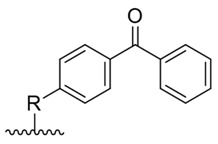	Silicon substrate	[67,68,74]
	Indium tin oxide coated glass	[69,77]
	Silica fibers	[70]
	Glass substrate	[71,72]
	Gold	[75,76,77,78,79]
	Stainless steel	[76,77]
	Glassy carbon	[77]
	Cellulose	[80]
	Cellulose nanocrystals	[81]
	Sodium montmorillonite Cloisite Na+	[82]
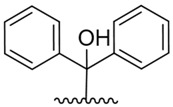	Polypropylene	[57,58,59,64,65]
	Cellulose acetate, poly (vinylidene)fluoride, polystyrene	[58]
	Poly(lactide acid)	[61]
Derived from BP via hydrogen abstraction from the surface according to Scheme 4	Poly(dimethylsiloxane)	[60]
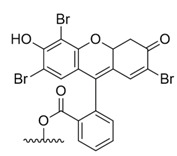	Silica nanoparticles	[87]
	Glass oligonucleotide biochip	[86]
	Cells	[88,89]
	Polyamine	[90]
	Cellulose	[91]
	Glass and silicon substrates	[85,92]
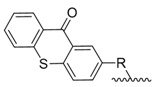	Silicon substrate	[96,97]
	Silica nanoparticles	[98,99]
	Polystyrene	[103]
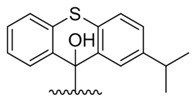	LDPE	[100,101,102]
Derived from thioxanthone via hydrogen abstraction from the surface according to Scheme 10		
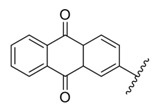	Poly(amid) fibers	[104]
	Cellulose	[105]
Synergist immobilization (tert. amine) for type II photoinitiators		
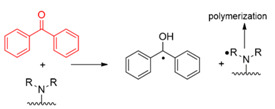	PP	[107]
	PET	[109]
	Hydrophilized PP	[106,108]
	Gold	[110,111]
	Silica nanoparticles	[112,113]

**Table 2:** Summary of surface coupled photoiniferters.

Photoiniferter	Substrate	Reference
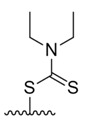	PS	[119,120]
	Glass substrate	[121]
	Poly(urethane)	[122]
	Cellulose membrane	[124]
	Cycloolefine substrate	[125]
	Silica microparticles	[123]
	Magnetic microspheres (Fe_3_O_4_)	[126]
	Hydrophilized PP	[127]
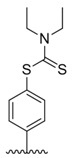	Silicon substrate	[128,129,130,131,132,133]
	Gold	[135,136,137,138,143]
	Iron oxide nanoparticles	[139,142]
	Aluminum nanoparticles	[140]

## Abbrevations

**Abbreviation**	**Meaning**
DMAEMA	2-(dimethylaminoethyl) methacrylate
AIBN	2,2-azobis(2-methylpropionitrile)
Irgacure 2959	2-hydroxy-4-(2-hydroxyethoxy)-2-methylpropiophenone
HEA	2-hydroxyethyl) acrylate
HEMA	2-hydroxyethyl) methacrylate
APTES	3-aminopropyltriethoxysilane
ACPA	4,4′-azobis(4-cyanopentanoic acid)
AAm	acrylamide
AA	acrylic acid
BP	benzophenone
BP-DS	benzophenone diazonium salt
BAPO	bis(acyl)phosphane oxide
CNC	cellulose nanocrystals
DC	dithiocarbamate
DTCA	dithiodiundecane- 11,1 diylbis[4({[(diethylamino) carbonothioyl] thioethyl) phenyl] carbamate}])]
GMA	glycidyl methacrylate
HAp	hydroxyapatite
HPTX	hyperbranched thioxanthone
LDPE	low-density-polyethylene
MAA	methacrylic acid
MMA	methyl methacrylate
SDBC	N-(diethylamino) dithiocarbamoylbenzyl-(trimethoxy)silane
DMAAM	N,N-dimethylacrylamide
NIPAM	N-isopropylacrylamide
NITEC	nitrile imine -mediated tetrazole-ene cycloaddition
PFPA	perfluorophenyl azides
PAH	photoacid
PAG	photoacid generator
PC	photoredox catalyst
PAA	poly (acrylic acid)
PMAA	poly (methacrylic acid)
PMMA	poly (methyl methacrylate)
PAAm	poly acrylamide
PEOX	poly(2-ethyl-2-oxazoline)
PDMAA	poly(dimethylacrylamide)
PEGMA	poly(ethylene glycol) methacrylate
PHEMA	poly(hydroxyethyl methacrylate)
PEI	poly(propyleneimine)
PEG	polyethylene glycol
PEGDA	polyethylene glycol diacrylate
PP	polypropylene
PS	polystyrene
DC-H	sodium- N,N diethyldithiocarbamate trihydrate
St	styrene
SI-ATRP	surface initiated atom transfer radical polymerization
SI-PET-RAFT	Surface Initiated Photoinduced Electron/Energy Transfer Reversible Addition-Fragmentation Chain Transfer Polymerization
SI-PIMP	surface initiated photoiniferter-mediated polymerization
SI-NMP	surface-initiated nitroxide-mediated polymerization
SI-RAFT	surface-initiated reversible addition fragmentation chain transfer
Two-photon absorption	TPA
Two-photon induced photopolymerization	TPIP
TX	thioxanthone
